# Evidence Map of Cochrane Reviews on Nutrition and Physical Activity in Healthy and At-Risk Populations: A Scoping Review

**DOI:** 10.1007/s10935-026-00924-8

**Published:** 2026-06-18

**Authors:** Karina Karolina De Santis, Katja Matthias, Knarik Nikoyan, Stefanie Maria Helmer

**Affiliations:** 1 Department of Prevention and Evaluation, Leibniz Institute for Prevention Research and Epidemiology – BIPS, Bremen, Germany; 2 Faculty of Health Service, Catholic University of Applied Sciences of North Rhine-Westphalia, Cologne, Germany; 3 Faculty 11 Human and Health Sciences, Working Group Evidence-Based Public Health, Institute of Public Health and Nursing Research, University of Bremen, Universitätsallee GW 1, 28359 Bremen, Germany

**Keywords:** Cochrane review, Nutrition, Physical activity, Scoping review

## Abstract

Cochrane reviews provide high-quality evidence syntheses. To guide the production and dissemination of new Cochrane reviews, an evidence map based on the characteristics of existing reviews is necessary. This study is a scoping review that aimed to create an evidence map of Cochrane reviews on nutrition and physical activity (PA) in healthy and at-risk populations, describe stakeholder involvement in review production and assess the dissemination of these reviews. This scoping review follows a registered protocol and adheres to the Preferred Reporting Items for Systematic Reviews and Meta-Analyses Extension for Scoping Reviews (PRISMA ScR) guideline. Eligible studies were Cochrane reviews on nutrition and PA for healthy and at-risk populations that were identified in the Cochrane Database of Systematic Reviews through to 22 January 2026 and selected independently by two researchers from 2043 search hits. Extracted data included bibliographic, population, and intervention or concept characteristics, information on stakeholder involvement in review production and open-access Altmetric data on the online and scientific attention towards Cochrane reviews. Data were processed into meaningful categories and synthesised using descriptive statistics. The included Cochrane reviews (*n* = 260) were published between 1999 and 2026. Review focus was on nutrition (*n* = 193, 74%), PA (*n* = 39, 15%), and nutrition + PA (*n* = 28, 11%). Most reviews included any populations by age and sex (*n* = 52, 20%) or specific populations by age (adults: *n* = 62, 24%; children: *n* = 54, 21%; and child-caregiver dyads: *n* = 50, 19%). The interventions or concepts were predominantly non-digital (*n* = 230, 88%), included mainly nutrition components (e.g. supplementation: *n* = 104, 40%; diet: *n* = 68, 26%), and were conducted in any unspecified settings (e.g. community, population; *n* = 226, 87%). The outcomes were mainly disease-related (e.g. disease prevention and risk factor reduction; *n* = 129, 50%) and reproductive (e.g. maternal and child health promotion; *n* = 56, 22%). There were less or no reviews with the following characteristics: (1) populations of older adults and males, (2) interventions or concepts with digital modality and with components targeting PA promotion, behaviour change, and other factors (e.g. policy), (3) specific settings (e.g. vulnerable, organisational), (4) outcomes targeting general health promotion and psychosocial (e.g. behavioural, cognitive, and mental) functioning. Involvement of various stakeholders (e.g. consumers, clinicians, and advisory boards) was reported in the production of 32% of Cochrane reviews. Online attention was highest for reviews with nutrition components (i.e. supplementation and diet) while scientific attention was highest for reviews with mixed components (i.e. PA and supplementation). New Cochrane reviews on nutrition and PA need to target specific populations (especially older adults and males) and settings, assess digital interventions or concepts, and focus on components and outcomes targeting physical and mental health promotion. Stakeholder involvement in review production and online and scientific attention could be further promoted to potentially improve review uptake in practice.

**Supplementary Information:**

The online version contains supplementary material available at https://doi.org/10.1007/s10935-026-00924-8.

## Background

Systematic reviews addressing topics on health promotion and disease prevention play a crucial role in not only guiding clinical practice, but also informing practice about effective public health interventions at the population level and support policy development (Lhachimi, 2020). Cochrane produces high-quality systematic reviews on any health-related topics for over 30 years (Higgins et al., 2024). Cochrane has recently changed its review production model and introduced the so-called thematic groups that will focus on specialised content based on current research priorities (Cochrane, 2022). One such group is the Cochrane Thematic Group ‘Nutrition and Physical Activity’ (Cochrane NaPA) (Cochrane, 2024; Wolfenden et al., 2024). The two main objectives of Cochrane NaPA are (1) to produce reviews that meet the current needs of relevant stakeholders (i.e. evidence users and decision makers) and (2) to maximise the use of evidence from such reviews by stakeholders via comprehensive dissemination and knowledge translation strategies (Wolfenden et al., 2024).

Due to the recent restructuring process within Cochrane, new topics can now be prioritised and evidence gaps in specific fields, such as interventions targeting obesity (Blaxall et al., 2024) can be identified specifically in the context of prevention. Furthermore, it is of importance not only to map future topics but also to examine stakeholder involvement in review production to determine if these topics meet the needs of practice (Nasser et al., 2026).

Hence, a systematic assessment of the past Cochrane reviews on nutrition and physical activity (PA) can be used to create an evidence map of topics that were already addressed in such reviews. The evidence map can then be used to identify evidence gaps that were not addressed in the existing reviews. Consequently, both the evidence map and gaps can be used as a basis to define research priorities for the production and dissemination of new reviews by the Cochrane NaPA. In line with the Cochrane NaPA objective 1, interventions or concepts and populations included in the past reviews need to be assessed to understand what evidence already exists. For example, the characteristics of such interventions or concepts could be described in terms of health focus (i.e. nutrition, PA, or both), components (e.g. diet or exercise for fall prevention), and outcomes (e.g. cardiovascular disease prevention). Due to the rapid advancement in digitalisation of health measures for health promotion and disease prevention (Jahnel et al., 2025), there is also a need to assess the modality of interventions or concepts (e.g. digital measures using smartphones vs. non-digital measures). Furthermore, since the focus of Cochrane NaPA is on measures that can contribute to health promotion and disease prevention on a population level (Wolfenden et al., 2024), the characteristics of healthy and at-risk populations (e.g. based on age and sex) need to be described.

In line with the Cochrane NaPA objectives 1 and 2, the dissemination and stakeholder involvement in review production also need to be assessed in the past reviews. Bibliographic research on Cochrane reviews suggests that while the online attention towards Cochrane reviews is high (De Santis et al., 2024; Rosengaard et al., 2025), the stakeholder involvement in review production is low (Helmer et al., 2023).

Stakeholders are individuals or groups involved in decision-making or evidence production in the field of health and healthcare, or those who's interests are directly or indirectly affected by such decisions (Cottrell et al., 2014; Petkovic et al., 2023). Stakeholders can contribute to multiple phases of the review process, including topic selection, preparatory meetings, and dissemination efforts (Grill, 2021). They may also be directly involved as part of the review team (Keown et al., 2008). Such engagement offers several important benefits. First, it helps ensure that research questions are directly aligned with the needs and priorities of the intended target groups, thereby increasing or confirming their relevance (Concannon et al., 2014). Second, stakeholder participation enhances transparency and the comprehensibility of the research process, thereby facilitating trust in the findings (Ray & Miller, 2017). Third, involving stakeholders in research and evidence synthesis can promote active knowledge translation, enabling evidence to be disseminated effectively to audiences that might not otherwise engage with academic publications (Keown et al., 2008). Stakeholders can also play a crucial role in reaching underrepresented or hard-to-reach populations by sharing findings through their own networks, thereby enhancing both the reach and acceptance of research outcomes (Esmail et al., 2015). However, stakeholder involvement in evidence generation is still low meaning that there is still a wide research-to-practice gap and an urgent need for research-stakeholder collaborations in health research (Wolfenden et al., 2022).

This study is a scoping review that aimed to create an evidence map of Cochrane reviews on nutrition and PA in healthy and at-risk populations, describe stakeholder involvement in review production and assess the dissemination of these reviews. The following research objectives were addressed in this scoping review:

1. to create an evidence map based on the bibliographic, population, intervention or concept, and outcome characteristics of Cochrane reviews on nutrition and PA,
2. to describe stakeholder involvement in review production,
3. to assess the dissemination of these reviews.

## Methods

### Protocol and Registration

This is a scoping review with a prospectively registered protocol (osf.oi/mqbnj). The reporting adheres to the Preferred Reporting Items for Systematic Reviews and Meta-Analyses Extension for Scoping Reviews (PRISMA ScR) guideline (Tricco et al., 2018). The PRISMA ScR checklist is reported in Supplementary file 1.

### Eligibility Criteria

The data source for this scoping review were Cochrane reviews. The eligibility criteria adhere to the PIOS (Population, Intervention, Outcome, Study type) framework and align with the Cochrane NaPA scope (Box 1 in Wolfenden et al., 2024). Included were Cochrane reviews on interventions or concepts targeting nutrition and PA for human populations of any age and sex who were healthy, at risk for clinical conditions, or mixed (healthy, at-risk, or with clinical conditions). Excluded were Cochrane reviews on interventions with other focus than nutrition and PA, with clinical or special populations (e.g. premature babies) in healthcare settings, and with published updates already included in this scoping review.

### Information Sources

The information source for this review was the Cochrane Database of Systematic Reviews.

### Search Strategy

The search syntax was developed by one researcher (KM) and calibrated within the team. The final search syntax included adjectives and medical subject headings (MeSH) for two main terms combined with the Boolean operator OR (i.e. ‘physical activity’ OR ‘nutrition’) and applied to titles, abstracts, and key words in Cochrane reviews published from database inception to search date (Supplementary file 2). The electronic search was conducted by one researcher (KM) on 14 May 2024 and updated on 22 January 2026. There were no language or date restrictions applied in the search. The results were stored in EndNote 20 (Clarivate).

### Study Selection

Study selection was done by two researchers (KM and SMH) independently using title, abstract, and full-text screening in Rayyan (Rayyan Systems, USA). The inter-rater agreement was not formally assessed and any disagreements were resolved by discussion between both researchers.

### Data Charting

Data charting was done by manually extracting data from review full-texts and review information on the Cochrane website and processing such data into quantitative categories. The categories were either pre-planned or inductively derived from the data. For example, review focus derived from review title and aim was classified into two pre-planned categories: ‘nutrition’ and ‘PA’. A third category was inductively detected in the data: ‘nutrition and PA’.

All data were extracted and processed by human researchers and artificial intelligence tools were not used in any aspect of this review. All researchers extracted different aspects of the data into a self-developed sheet in Excel 10 (Microsoft Inc.). To reduce bias in data extraction, two researchers (KKDS, KM) cross-checked all processed data, discussed any inconsistencies, and reached consensus by discussion.

### Variables (Data Items) and Data Processing

The variables extracted from Cochrane reviews were organised into four groups:

1. bibliographic characteristics: publication year, author region, review type, studies in review, review focus obtained from review title and aim (nutrition, PA, nutrition and PA).
2. population characteristics: sex, age group.
3. intervention or concept characteristics: component (e.g. supplementation, PA promotion); outcome (e.g. disease prevention); modality (non-digital or digital, e.g. interventions delivered via smartphones or other devices with internet access or remote, e.g. delivered via telephone or any media); setting (any or specific, e.g. workplace).
4. dissemination characteristics: stakeholder involvement in review production (e.g. advisory board, consumer representative, or other stakeholders, e.g. clinicians mentioned in Cochrane review text, e.g. in acknowledgements); online dissemination based on open-access Altmetric data available for each review on the Cochrane website on 23 February 2026.

Altmetric data were used to quantify the online and scientific attention towards each Cochrane review. Online attention was measured using Altmetric Attention Score that is a weighted number of mentions of a publication in various online channels (e.g. Wikipedia) (Altmetric, 2025). Scientific attention was measured using Dimensions Badge (i.e. Dimensions Citations Score) that is a total number of citations of a publication in the sources indexed by Dimensions database (Dimensions, 2018). The coverage of Dimensions database in health and medical sciences highly overlaps with other international bibliographic databases, including SCOPUS (86%) and Web of Science (89%) (Martín-Martín et al., 2021).

### Critical Appraisal

Critical appraisal was not conducted because this scoping review did not aim to evaluate the effectiveness of interventions. Furthermore, Cochrane reviews have similar quality because they adhere to standardised methodological guidelines for review production and reporting.

### Data Synthesis

The data were synthesised using descriptive statistics (i.e. absolute and relative frequencies) in Excel and IBM-SPSS 29. The focus of data synthesis was on creating an evidence map based on bibliographic and review characteristics (i.e. review focus, population, intervention, and outcome) and identifying evidence gaps based on the review characteristics stratified by review focus.

### Dissemination

We planned to disseminate the study results using the following strategies: conference abstract or article in a peer-reviewed academic journal, and plain language summaries in English and in German (Additional file 5).

## Results

### Study Selection

**Fig. 1:**
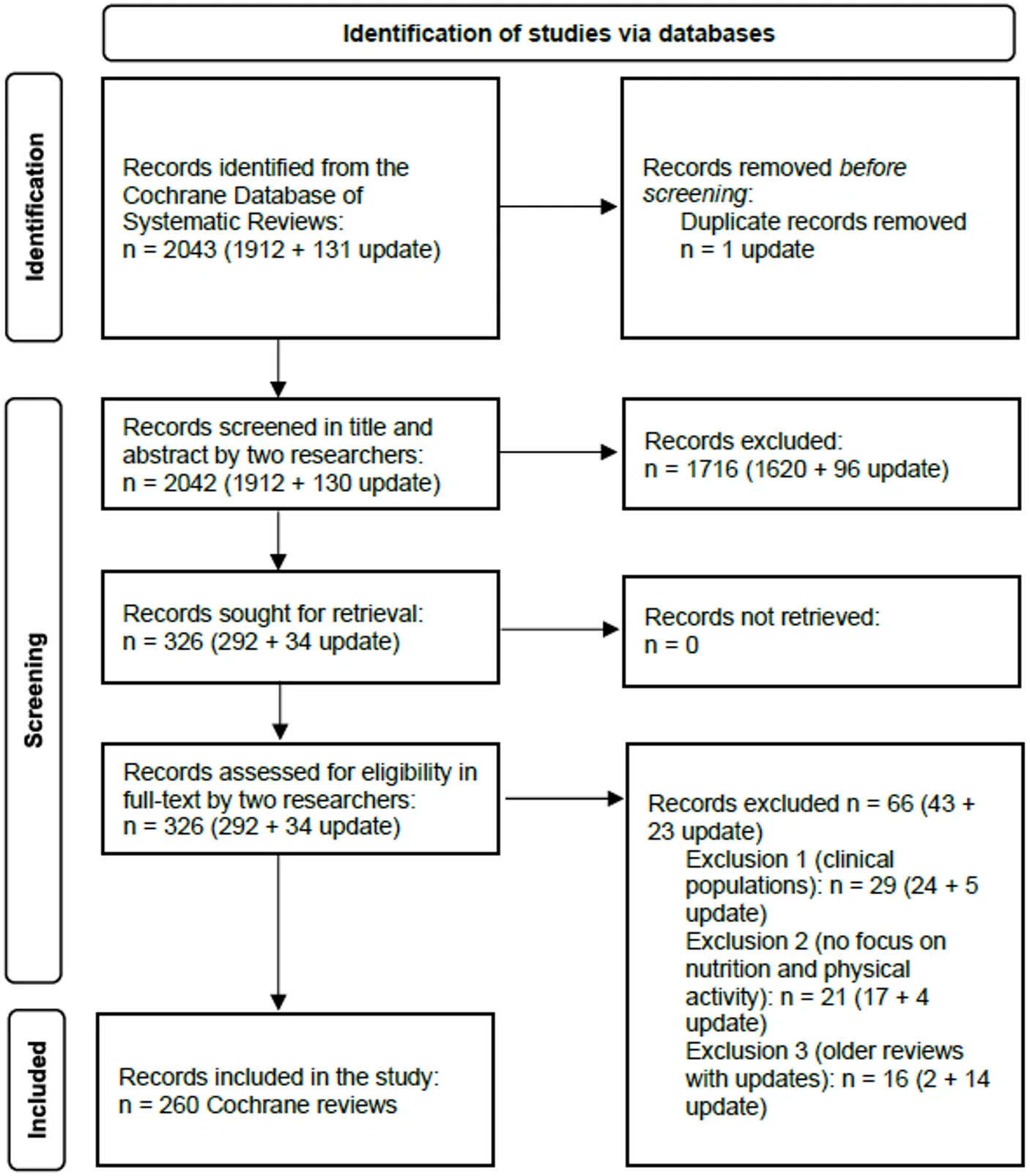
Study selection (PRISMA-ScR flowchart)

Among 2043 sources identified in the electronic search, 1716 were excluded in the title and abstract screening and 66 were excluded in the full-text screening (Fig. 1, Supplementary file 3). The remaining 260 Cochrane reviews were included in this scoping review (Supplementary file 4).

### Bibliographic Characteristics of the Included Studies (*n* = 260 Cochrane Reviews)

The 260 Cochrane reviews were systematic reviews (98%) and overviews of reviews (2%; Table 1). The reviews were published between 1999 and 2026 and originated predominantly from Europe, North America, and Australia and Oceania. Overall, 98% (*n* = 254) of reviews included at least one study (i.e. between 1 and 195 studies), while 2% (*n* = 6) of reviews did not include any studies. Most reviews had nutrition focus (74%). Less reviews had PA (15%) and nutrition and PA (11%) focus.

### Population, Intervention or Concept, and Outcome Characteristics

Most reviews included populations of either any age or sex (20%) or of different age groups (adults in 24%, children in 21%, and child-caregiver dyads in 19% of reviews; Table 2). Less reviews included older people of any sex (6%) and females of any age (8%) and no reviews targeted males of any age.

**Table 1:** Bibliographic characteristics and review focus in 260 Cochrane reviews

Characteristics	Minimum	Maximum
Publication year	1999	2026
Included studies	0	195
	*n* = 260	**% of 260**
Reviews by included studies		
Reviews with 1-195 studies	254	97.69
Reviews with 0 studies	6	2.31
Author region		
Europe	118	45.38
North America	45	17.31
Australia and Oceania	43	16.54
Asia	32	12.31
Africa	13	5.00
South America	9	3.46
Review type		
Systematic review	256	98.46
Overview of reviews (i.e. umbrella review)	4	1.54
Review focus derived from review title and aim		
Nutrition	193	74.23
Physical activity (PA)	39	15.00
Nutrition and PA	28	10.77

Intervention or concept components in most reviews were related to nutrition (supplementation in 40%, diet in 26%, and fortification of food in 7% of reviews; Table 2). Less reviews included PA promotion (22%), behavioural (18%) and other components (12%).

Intervention or concept modality in most reviews (88%) was non-digital (e.g. dietary advice or physical exercises via in-person or group consultations and meetings; Table 2). Less reviews (9%) included digital interventions or concepts with internet-based (e.g. via smartphones) or remote (e.g. via telephone or media) delivery.

Most reviews (87%) addressed any settings (Table 2). The minority of reviews included specific settings (vulnerable populations in 5%, care in 3%, and organisation settings in 2% of reviews).

**Table 2:** Populations and interventions or concepts in 260 Cochrane reviews

Characteristics	*n* = 260	% of 260
Not reported (reviews with no included studies)	6	2.31
Reported	254	97.69
Population		
Any age or sex	52	20
Specific age (any sex)		
Adults	62	23.85
Children	54	20.77
Dyads (child-caregivers)	50	19.23
Older adults	16	6.15
Specific sex (any age)		
Female	20	7.69
Male	0	0
Intervention or concept: Component (one or multiple per review)		
Supplementation	104	40.00
Diet	68	26.15
Fortification	17	6.54
Physical activity (PA) promotion	58	22.31
Behavioural (e.g. education or advice to target behaviour change)	48	18.46
Other (e.g. policy, food availability, taxation)	32	12.31
Intervention or concept: Modality		
Non-digital	230	88.46
Digital (alone or digital and non-digital)	24	9.23
Intervention or concept: Setting		
Any unspecified (e.g. community, population)	226	86.92
Specific		
Vulnerable (e.g. geographic region, low-middle income countries, ethnic groups, low socioeconomic status)	14	5.38
Care (e.g. childcare, aged care, healthcare)	9	3.46
Organisation (e.g. sport, workplace)	5	1.92

Most reviews assessed outcomes that were predominantly disease-related (50%) and reproductive (22%; Table 3). Less reviews assessed health promotion (13%), functional and development (8%) and psychosocial (5%) outcomes.

**Table 3:** Outcomes in 260 Cochrane reviews

Characteristics	*n* = 260	% of 260
Not reported (reviews with no included studies)	6	2.31
Reported	254	97.69
Outcome types		
Disease-related (i.e. disease prevention, risk factor reduction)	129	49.62
Reproductive (i.e. maternal and child health promotion)	56	21.54
Health promotion (i.e. healthy lifestyle promotion)	33	12.69
Functional and development (e.g. balance, fall prevention, growth)	22	8.46
Psychosocial (i.e. behavioural, cognitive and mental functioning promotion)	14	5.38
Outcome subtypes		
Disease-related outcomes		
Cardiovascular	35	13.46
Infection	24	9.23
Overweight and obesity	18	6.92
Vitamin and micronutrient deficiencies	13	5.00
Nutrition status and malnutrition	10	3.85
Allergy, asthma	9	3.46
Anaemia	3	1.15
Cancer	6	2.31
Any (e.g. mortality, morbidity, non-communicable disease)	6	2.31
Eye disease	3	1.15
Diabetes	2	0.77
Reproductive outcomes		
Maternal and child health	41	15.77
Feeding assistance	9	3.46
Gestational diabetes prevention	6	2.31
Health promotion outcomes		
Physical activity (PA) promotion	15	5.77
Healthy diet promotion	11	4.23
Healthy lifestyle promotion	7	2.69
Functional and development outcomes		
Growth	10	3.85
Balance, fractures, falls	7	2.69
Musculoskeletal pain	5	1.92
Psychosocial outcomes		
Behavioural, cognitive and mental functioning promotion	14	5.38

### Review Characteristics by Review Focus

**Fig. 2:**
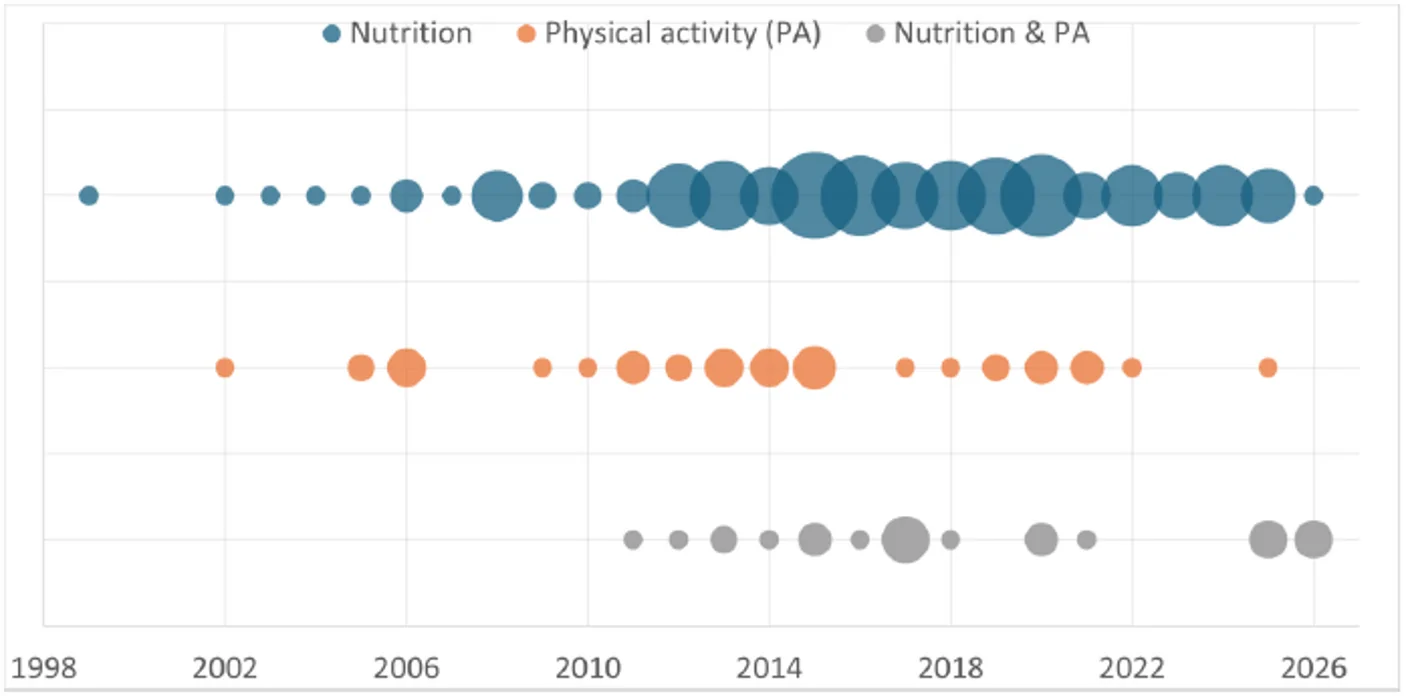
Review focus by publication year in 260 Cochrane reviews. Bubble size indicates the number of reviews (smallest bubble = 1 review, largest bubble = 20 reviews); intermediate sizes are scaled proportionally

The stratification by review focus revealed that the reviews were published between 1999 and 2026 (nutrition focus), 2002–2025 (PA focus), and 2011–2026 (nutrition and PA focus; Fig. 2).

**Fig. 3:**
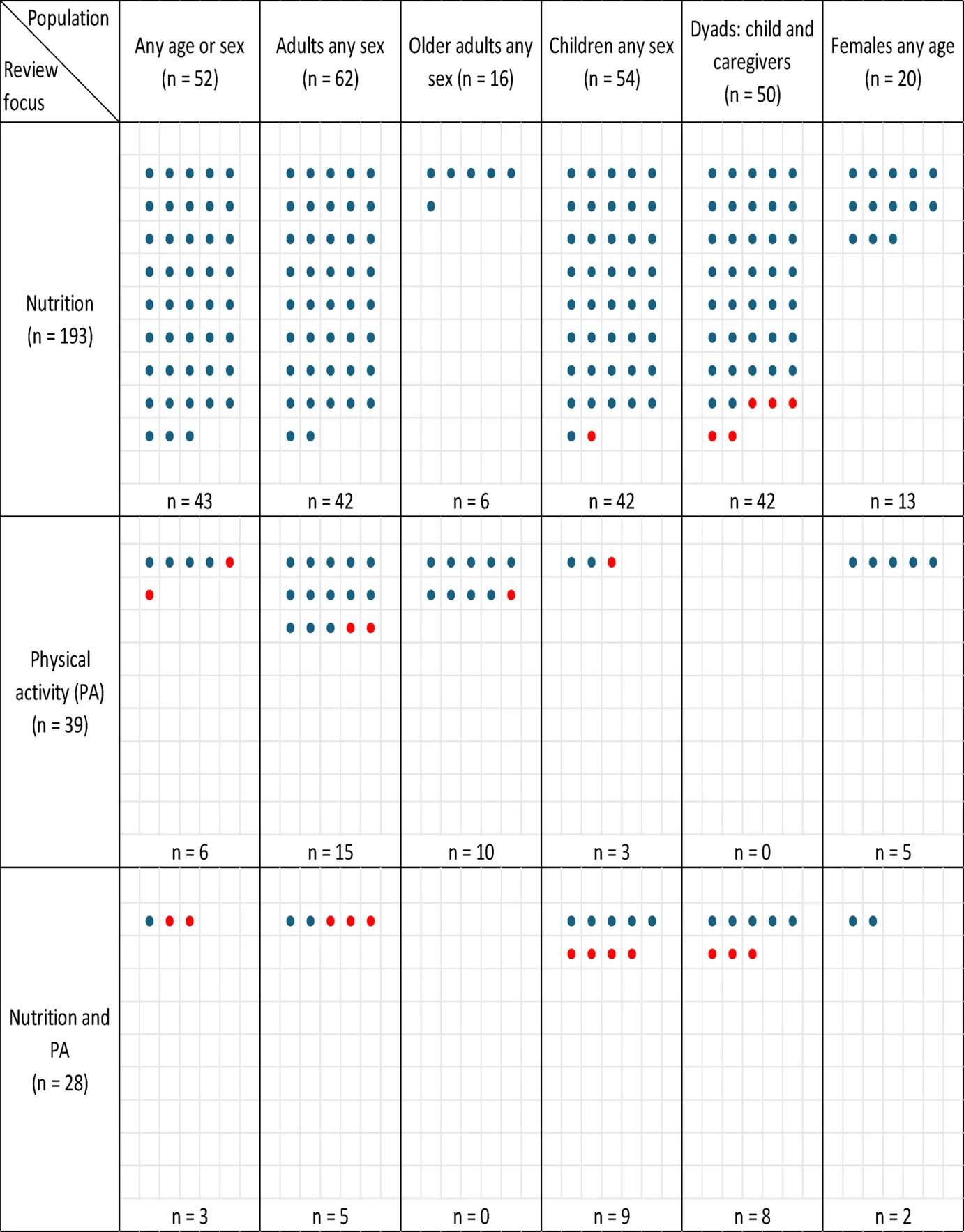
Review focus by population and modality of interventions or concepts in 260 Cochrane reviews. The dots represent *n* = 254 Cochrane reviews with population and intervention or concept modality. The *n* = 6 reviews (*n* = 5 with nutrition focus and *n* = 1 with nutrition and PA focus) without population and modality are not shown. Legend: blue dots = reviews with non-digital modality, red dots = reviews with digital (alone or digital and non-digital) modality

The populations differed by review focus (nutrition focus: mainly any population, adults, children, and dyads; PA focus: mainly adults and older adults; nutrition and PA focus: mainly children and dyads; Fig. 3). Less or no reviews included older people (nutrition, nutrition and PA focus) and females (any focus).

Most reviews with any focus included non-digital interventions or concepts (Fig. 3). Digital modality was included predominantly in reviews with nutrition and PA focus. Less or no reviews with any focus included digital interventions or concepts for older adults and females.

**Fig. 4:**
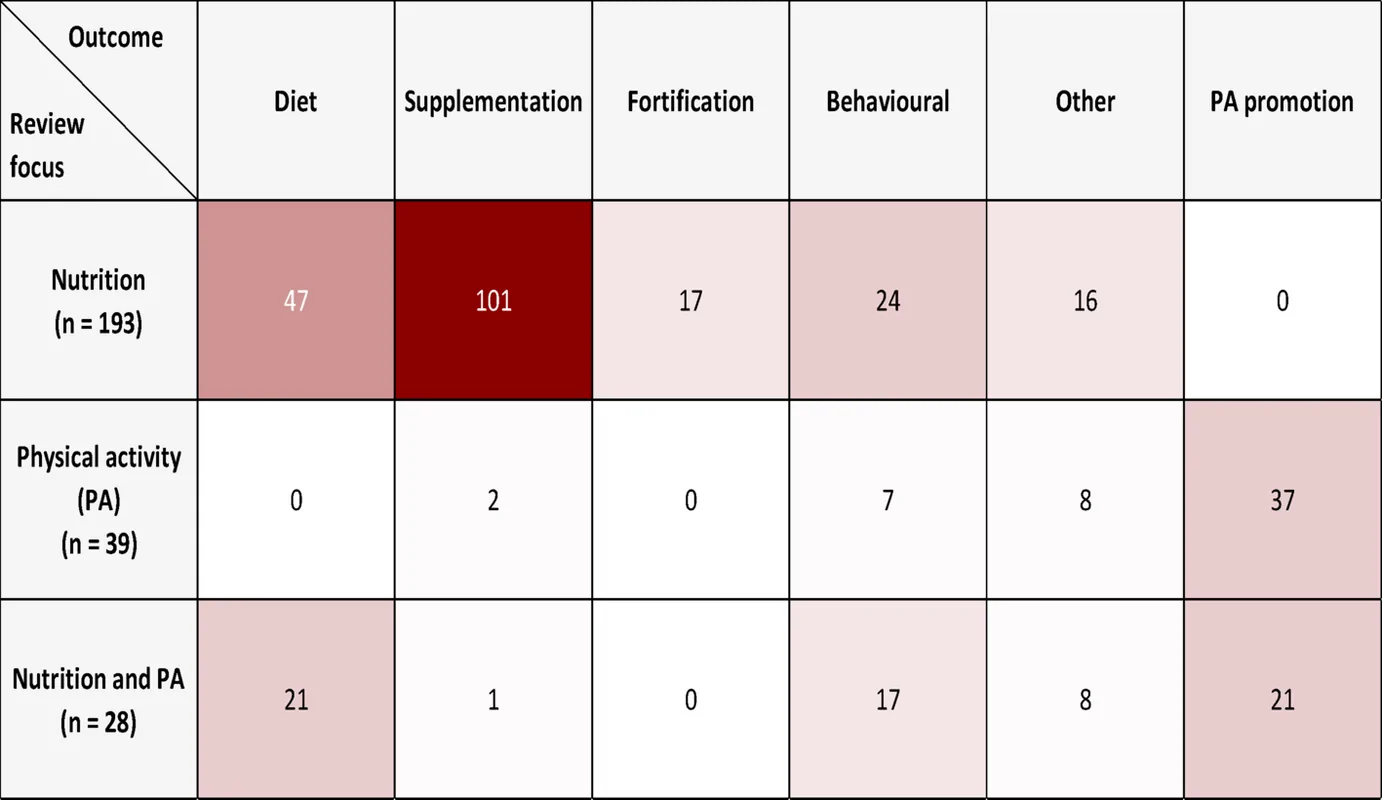
Review focus by intervention or concept component in 260 Cochrane reviews. The numbers represent intervention or concept components in *n* = 254 Cochrane reviews. The *n* = 6 reviews (*n* = 5 with nutrition focus and *n* = 1 with nutrition and PA focus) without components are not shown. The total number exceeds 254 because some reviews included multiple components

Most intervention or concept components included supplementation (nutrition focus), diet (nutrition, nutrition + PA focus), and PA promotion (PA, nutrition + PA focus; Fig. 4). Less or no reviews with PA and nutrition and PA focus included fortification, behavioural and other components.

Most reviews with any focus addressed any setting (Table 4). Less or no reviews with any focus addressed specific settings, including vulnerable populations, care and organisational settings.

**Table 4:** Review focus by setting in 260 Cochrane reviews

Characteristic	*n* = 260	% of 260
Not reported (reviews with no included studies)	6	2.31
Reported	254	97.69
Setting		
Nutrition		
Any	173	66.54
Care	3	1.15
Vulnerable	12	4.62
Organisation	0	
Physical activity (PA)		
Any	32	12.31
Care	3	1.15
Vulnerable	0	
Organisation	4	1.54
Nutrition and PA		
Any	21	8.08
Care	3	1.15
Vulnerable	2	0.77
Organisation	1	0.38

**Fig. 5:**
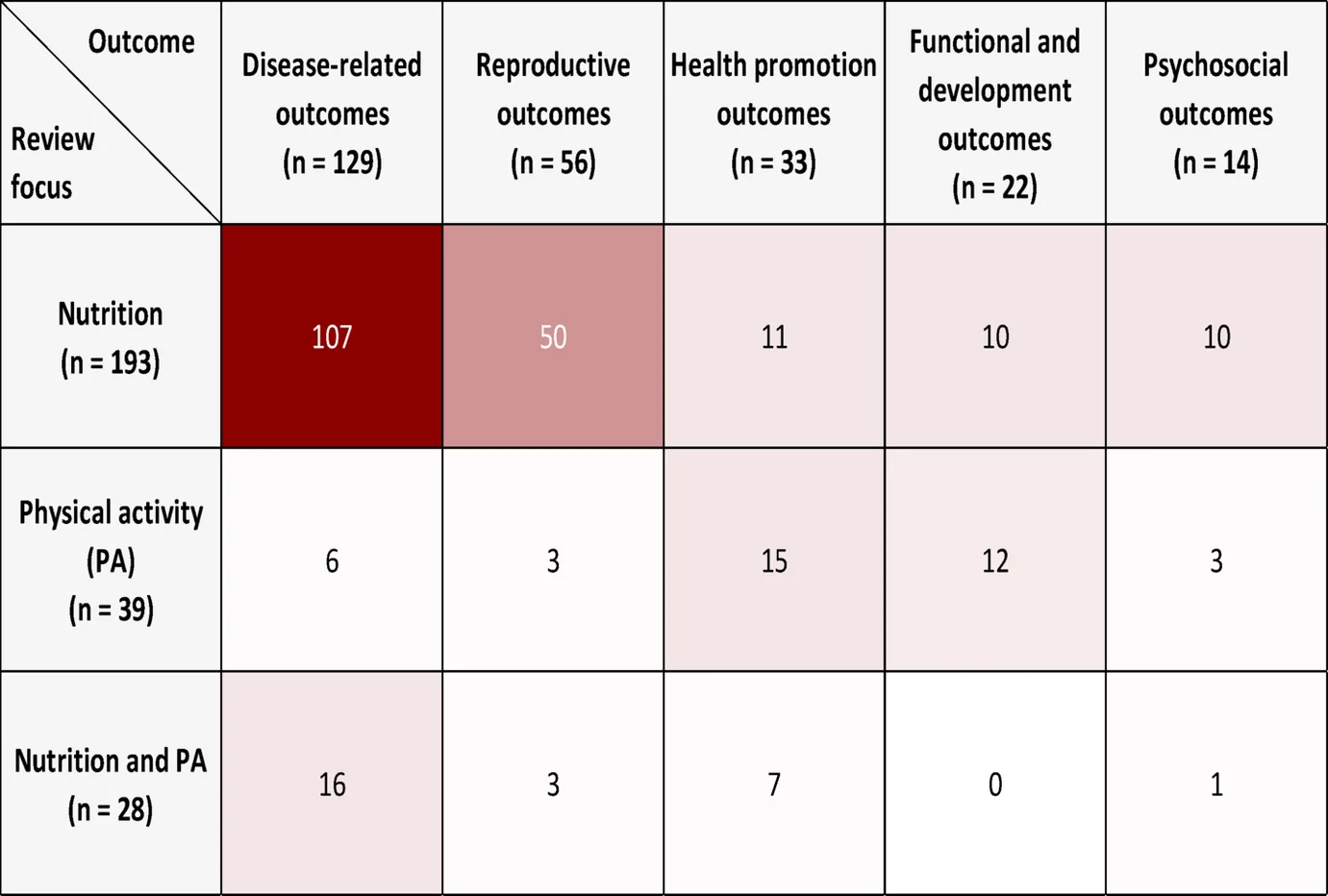
Review focus by outcome in 260 Cochrane reviews. The numbers represent *n* = 254 Cochrane reviews with outcomes. The *n* = 6 reviews (*n* = 5 with nutrition focus and *n* = 1 with nutrition and PA focus) without outcomes are not shown

The outcomes differed by review focus. Most outcomes were disease-related and reproductive (nutrition focus; Fig. 5). Less or no reviews with any focus included health promotion, functional and development, and psychosocial outcomes (Fig. 5).

### Stakeholder Involvement in Review Production

Overall, 32% of reviews reported that various stakeholder groups were involved in review production (Table 5). These included predominantly consumers (in 23% of reviews) and other stakeholders (in 16% of reviews). Less reviews (6%) included advisory boards.

**Table 5:** Stakeholder involvement in review production in 260 Cochrane reviews

Characteristics	*n* = 260	% of 260
Stakeholder involvement		
Yes	84	32.31
No or no information	176	67.69
Stakeholder groups (one or multiple groups per review)		
Consumers	60	23.08
Other (e.g. clinicians, health organisation representatives)	42	16.15
Advisory board	16	6.15

Among reviews with consumer involvement, the consumers in 28/60 reviews were recruited from the international panel of the Cochrane Pregnancy and Childbirth Group and contributed to the pre-publication editorial process alongside peer reviewers and statistical advisers. The other stakeholders included representatives from the World Health Organisation who acted as commissioning bodies, funders, or contributors to design, conduct, and interpretation of review findings. In one review (Mosdøl et al., 2017) national health institutions not only advised but also actively contributed to shaping the content and focus of the review. The advisory board typically contributed to protocol development, review design and interpretation of findings. One review (Lhachimi et al., 2020) mentioned an interdisciplinary advisory board consisting of policymakers, researchers, and practitioners that was actively involved throughout several stages of the review process.

### Online Dissemination

The online and scientific attention towards the 260 Cochrane reviews followed similar patterns (Table 6). The Altmetric Attention Scores (online attention) ranged between 1 and 4758 (median of 46). The Dimensions Citations Scores (scientific attention) ranged between 1 and 2917 (median of 89).

**Table 6:** Online and scientific attention towards 260 Cochrane reviews according to Altmetric data

Characteristics	Online attention: Altmetric Attention Score		Scientific attention: Dimensions Citations Score	
All scores (*n*)	*n* = 260		*n* = 260	
Minimum-maximum	1-4758		1-2917	
Median	46		89	
Top 25% of scores (n)	*n* = 65		*n* = 64	
Minimum-maximum	109–4758		166–2917	
Median	268		317	
Publication year	2006–2025		2004–2021	
Review focus	*n* = 65	**% of 65**	*n* = 64	**% of 64**
Nutrition	49	75.38	37	57.81
Physical activity (PA)	12	18.46	17	26.56
Nutrition and PA	4	6.15	10	15.63
Population				
Adults (any sex)	23	35.38	17	26.56
Any (age or sex)	17	26.15	12	18.75
Older adults (any sex)	9	13.85	11	17.19
Children (any sex)	6	9.23	7	10.94
Dyads (child-caregivers)	6	9.23	11	17.19
Female (any age)	4	6.15	6	9.38
Intervention or concept component				
Supplementation	25	38.46	22	34.38
Diet	23	35.38	18	28.13
Fortification	2	3.08	0	0.00
PA	16	24.62	25	39.06
Behavioural	8	12.31	10	15.63
Other	8	12.31	9	14.06
Intervention or concept modality				
Non-digital	64	98.46	61	95.31
Digital	1	1.54	3	4.69
Intervention or concept setting				
Any unspecified	65	100.00	62	96.88
Specific	0	0	2	3.13
Outcome				
Disease-related outcomes	37	56.92	29	45.31
Cardiovascular	14	21.54	11	17.19
Infection	12	18.46	4	6.25
Overweight, obesity	6	9.23	5	7.81
Functional and development outcomes	8	12.31	7	10.94
Balance, fractures, falls	6	9.23	6	9.23
Reproductive outcomes	8	12.31	12	18.75
Maternal and child health	6	9.23	9	14.06
Psychosocial outcomes	7	10.77	4	6.25
Health promotion outcomes	5	7.69	12	18.75

Altmetric data for each review were extracted from the Cochrane website on 23 February 2026

Overall, *n* = 65 reviews had the top 25% of online attention scores (i.e. Altmetric Attention Scores between 109 and 4758, median of 268) and were published in 2006–2025. Overall, *n* = 64 reviews had the top 25% of scientific attention scores (i.e. Dimensions Citations Scores between 166 and 2917, median of 317) and were published in 2004–2021.

Among the reviews with the top 25% of online and scientific attention scores (Table 6), most reviews had nutrition focus and included adults or any populations, interventions or concepts with non-digital modality, any unspecified settings, and disease-related outcomes (mainly prevention of cardiovascular disease, infection, overweight and obesity). However, online attention was highest for reviews with nutrition components (i.e. supplementation and diet) while scientific attention was highest for reviews with mixed (i.e. PA and supplementation) components.

## Discussion

### Summary of Main Findings

This scoping review mapped the characteristics of 260 Cochrane reviews on nutrition and PA published up to 2026. While there is already extensive research in the areas of nutrition and PA covered by Cochrane reviews, the evidence gaps identified here could be considered as topics for future evidence syntheses by the Cochrane Thematic Group NaPA or other non-Cochrane reviews. Specifically, further reviews on nutrition and PA could target (1) populations of older adults and males, (2) interventions or concepts with digital modality and with components focusing on PA promotion, behaviour change, and other factors (e.g. policy), (3) specific settings (e.g. vulnerable, organisational), and (4) outcomes related to general health promotion and psychosocial (e.g. behavioural, cognitive, and mental) functioning. Stakeholder involvement in review production and online and scientific attention could be further promoted to potentially improve review uptake in practice.

### Evidence Gaps in the Field of Nutrition and Physical Activity

Only 16 (6%) of Cochrane reviews specifically included older adults, with 10 of these reviews focusing on PA and six on nutrition. Fall prevention and the strengthening of cognitive and mental functioning were addressed in these reviews as well as in other research (Sun et al., 2023; Taylor et al., 2021). Future reviews could aim to assess population-based approaches and evaluate the long-term effects of programs on this level (Izquierdo et al., 2021; Taylor et al., 2021). In the field of nutrition, interventions for older adults mostly consisted of the supplementation of specific nutritional components like vitamins or omega-3. Even though malnutrition and nutritional deficiencies are known health complaints in older people, other systemic diseases such as metabolic syndrome are of underestimated importance (Volkert et al., 2024). Thus, more comprehensive dietary programs are needed, such as interventions based on behaviour change techniques, but these are less common and often do not describe their implementation strategies (Zaslavsky et al., 2021).

Reviews on older adults could focus on specific settings, since community venues, health, social and long-term care settings as well as the workplace are recommended to enhance the provision of preventive interventions (WHO, 2018) and show promising impact across diverse locations (Pinheiro et al., 2022). Therefore, it is also necessary to increasingly investigate interventions in specific settings to demonstrate relevant action needs.

Overall, few gender-specific prevention measures were identified, apart from studies on reproductive health. It is well established that sex-specific differences in cardiovascular risk factors, such as a poor diet and low physical activity, exist, which underscores the need for tailored prevention strategies that address the unique risk profiles of males and females (Kubielas et al., 2025). Overall, research shows that males are less likely to participate in health promotion and preventive interventions, as they often view them as a ‘women’s issue’ (Howell et al., 2023) and are also less likely to participate in research in this area (Sharp et al., 2020). More evidence on health promotion and disease prevention targeting specifically males is needed to address health issues relevant for this biological sex group.

Despite the high interest in digital technology use in public health interventions and concepts (Jahnel et al., 2025), only 9% of Cochrane reviews on nutrition and PA included studies with digital solutions. Such reviews typically focused on objective measurement of PA using digital devices. Other non-Cochrane reviews on digital interventions in public health highlight the challenges in evaluating the effects of digital interventions and in promoting the adherence and engagement with such interventions (Alley et al., 2024; De Santis et al., 2022; Singh et al., 2024). Therefore, using alternative and agile methods for evaluation of digital interventions is recommended (Michie et al., 2017) but rarely implemented in prevention research (Hrynyschyn et al., 2022). Future reviews could focus on the outcome evaluation of digital interventions using such alternative methods. Furthermore, evidence syntheses are needed to investigate digital technology use in public health by specific population groups, cultural differences in such use (Singh et al., 2024), and social inequalities and factors that contribute to digital divide beyond age and sex (König et al., 2025).

### Dissemination and Stakeholder Involvement

The reviews with the highest online and scientific attention predominantly focused on adult populations and broad health outcomes. A bibliometric study based on Altmetric data of Cochrane reviews also revealed that adult and female health but also exercise and diet were popular keywords used by authors (Kolahi, 2020). In this study, the Altmetric data indicate that Cochrane reviews on nutrition and PA are frequently mentioned online and also cited in scientific sources. However, more research is needed on how the recipients of the online information on the existence of Cochrane reviews (e.g. social media users) interact with or use such information (De Santis et al., 2024).

Just 32% of the Cochrane reviews reported any form of stakeholder involvement. This is consistent with 34% reported by Helmer et al. (2023) in a bibliographic analysis of Cochrane Public Health reviews, suggesting that limited reporting of such engagement may be a persistent issue across thematic areas. Similar to these findings (Helmer et al., 2023), we also observed that stakeholder roles were most often linked to protocol development, review design, and interpretation of results in Cochrane reviews on nutrition and PA. Even in rapid reviews, which are particularly relevant for political decision making due to their timely manner, there is a low level of stakeholder engagement (Nussbaumer-Streit et al., 2025).

Our analysis also revealed that the depth and nature of stakeholder involvement were often only briefly described, echoing the conclusions of Pollock et al. (2018), who noted a general lack of detailed reporting on this aspect in systematic reviews (Pollock et al., 2018). Without such detail, it is difficult to assess whether engagement was meaningful or merely procedural. Nevertheless, positive examples in our dataset demonstrate that sustained, multi-stage collaboration with diverse stakeholder groups is both feasible and valuable. For example, in one Cochrane review (Lhachimi et al., 2020), an interdisciplinary advisory board consisting of policymakers, researchers, and practitioners was established and actively involved throughout several stages of the review process. Another Cochrane review (Mosdøl et al., 2017) reported that national health institutions not only advised but also actively contributed to shaping the content and focus of the review. Furthermore, extensive engagement, including input from institutions such as the WHO and the Cochrane Consumers and Communication Group, alongside contributions from field experts and consumer representatives were also mentioned (Palmer et al., 2020). Given the established benefits of stakeholder engagement, such as ensuring the relevance of research questions, enhancing transparency, facilitating dissemination, and improving uptake in practice, the low rate of reported stakeholder involvement in our study suggests a missed opportunity for maximising the impact of Cochrane reviews on nutrition and PA. Optimising and broadening stakeholder recruitment to include underrepresented groups and those most affected by nutrition and PA interventions and concepts, may help ensure that future reviews address the needs of all relevant populations. Frameworks such as Cochrane’s ACTIVE (Authors and Consumers Together Impacting on Evidence) initiative offer a structured approach to defining and reporting stakeholder involvement and could help authors plan for engagement that is both systematic and transparent (Pollock et al., 2019). Complementary to this, the reflections provided by Brown et al. (2023) underline practical strategies for supporting meaningful involvement, such as early engagement in question formulation, regular communication, and shared decision-making processes (Brown et al., 2023). Future Cochrane reviews could benefit from integrating stakeholder engagement as a planned methodological component rather than as an optional add-on. This may involve adopting existing frameworks, allocating resources and time for meaningful participation, and ensuring that reporting meets emerging standards for transparency. Strengthening these practices would align with Cochrane NaPA objectives and could enhance the societal relevance and uptake of review findings.

### Limitations

The strength of this scoping review is the high number of included Cochrane reviews and the identification of research gaps that could be addressed in future Cochrane reviews on nutrition and PA. However, there were also several limitations in this scoping review. First, this scoping review included Cochrane reviews alone and thus is biased towards topics addressed by these reviews. Meta-research approach could be used to identify research priorities for future Cochrane reviews based on topics addressed in systematic reviews published in other journals. Second, the evidence map obtained in this scoping review suggests that some topics were rarely or not addressed in the Cochrane reviews on nutrition and PA. The reasons for such evidence gaps should be further assessed. In general, evidence gaps can result from the lack of evidence in primary studies or from a focused coverage by Cochrane reviews. Third, supplementation and fortification of food were considered as part of nutrition in this scoping review although they were not identified among the priority topics by the Cochrane NaPA group (Wolfenden et al., 2024). Fourth, this scoping review did not investigate if the interventions or concepts improved any health outcomes. Future systematic reviews could focus on the efficacy of more specific interventions or concepts in narrower populations (e.g. older people or males) as identified in this scoping review.

### Implications for Research and Prevention Practice

Numerous Cochrane reviews in the areas of nutrition and PA have already been conducted providing a strong research base for prevention practice. However, evidence gaps have been identified that can be closed by future reviews to better inform the development of evidence-based prevention measures. Cochrane reviews targeting specific socio-demographic subgroups and digital interventions or concepts in the field of nutrition and PA could be conducted in the future. Review authors could use existing open-source frameworks to set research priorities together with stakeholders (Hoekstra & Lhachimi, 2025). These can help to achieve the maximum practical benefit of research.

The dissemination and collaboration of stakeholders throughout the review process, ideally from the planning stage onwards, can contribute to research being more closely aligned with practice and ultimately leading to evidence-informed interventions being implemented in practice. Overall, it appears to be necessary to move beyond conducting only evidence syntheses and to systematically develop evidence-informed implementation strategies. Studies investigating how interventions can be successfully implemented in practice are currently scarce and offer much potential for evidence-based prevention (Wolfenden et al., 2022).

## Conclusions

This scoping review mapped the characteristics of 260 Cochrane reviews on nutrition and PA published up to 2026. While there is already extensive research in the areas of nutrition and PA covered by Cochrane reviews, the evidence gaps identified here could be considered as topics for future evidence syntheses by the Cochrane Thematic Group NaPA or other non-Cochrane reviews. New reviews on nutrition and PA need to target specific populations (especially older adults and males) and settings, assess digital interventions or concepts, and focus on components and outcomes targeting physical and mental health promotion. Stakeholder involvement in review production and online and scientific attention could be further promoted to potentially improve review uptake in practice.

## Supplementary Information

Below is the link to the electronic supplementary material.

Supplementary file 1: PRISMA-ScR Checklist. Supplementary file 2: Search strategy. Supplementary file 3: List of excluded studies. Supplementary file 4: List of included studies. Supplementary file 5: Plain language summary

## Data Availability

The dataset supporting the conclusions of this article can be requested from the corresponding author.

## Abbreviations

MeSH: Medical subject heading
Cochrane NaPA: Cochrane Thematic Group ‘Nutrition and Physical Activity’
PA: Physical activity
PIOS: Population, intervention, outcome, study type
PRISMA ScR: Preferred reporting items for systematic reviews and meta-analyses extension for scoping reviews
