# Supplementary material for: Evidence Map of Cochrane Reviews on Nutrition and Physical Activity in Healthy and At-Risk Populations: A Scoping Review
Source: J Prev (2022). 2026 Jun 18;47(6):1033–56. doi: 10.1007/s10935-026-00924-8 (PMC13759555; doi:10.1007/s10935-026-00924-8)
Supplement: Supplementary file 1 [file 10935_2026_924_MOESM1_ESM.pdf]

Appendix
PRISMA-ScR Checklist

Section	Item	PRISMA-ScR Checklist Item	Fulfilled / Location
Title	1	Identify the report as a scoping review.	Title
**Abstract**			
Structured summary	2	Provide a structured summary that includes (as applicable): background, objectives, eligibility criteria, sources of evidence, charting methods, results, and conclusions that relate to the review questions and objectives.	Abstract
**Introduction**			
Rationale	3	Describe the rationale for the review in the context of what is already known. Explain why the review questions/objectives lend themselves to a scoping review approach.	Background
Objectives	4	Provide an explicit statement of the questions and objectives being addressed with reference to their key elements (e.g., population or participants, concepts, and context) or other relevant key elements used to conceptualize the review questions and/or objectives.	Background, last paragraph
**Methods**			
Protocol and registration	5	Indicate whether a review protocol exists; state if and where it can be accessed (e.g., a Web address); and if available, provide registration information, including the registration number.	Methods, Protocol and registration
Eligibility criteria	6	Specify characteristics of the sources of evidence used as eligibility criteria (e.g., years considered, language, and publication status), and provide a rationale.	Methods, Eligibility criteria
Information sources	7	Describe all information sources in the search (e.g., databases with dates of coverage and contact with authors to identify additional sources), as well as the date the most recent search was executed.	Methods, Information sources
Search	8	Present the full electronic search strategy for at least 1 database, including any limits used, such that it could be repeated.	Methods, search strategy, Supplementary file 2

Selection of sources of evidence	9	State the process for selecting sources of evidence (i.e., screening and eligibility) included in the scoping review.	Methods, study selection
Data charting process	10	Describe the methods of charting data from the included sources of evidence (e.g., calibrated forms or forms that have been tested by the team before their use, and whether data charting was done independently or in duplicate) and any processes for obtaining and confirming data from investigators.	Methods, data charting
Data items	11	List and define all variables for which data were sought and any assumptions and simplifications made.	Methods, variables (data items) and data processing
Critical appraisal of individual sources of evidence	12	If done, provide a rationale for conducting a critical appraisal of included sources of evidence; describe the methods used and how this information was used in any data synthesis (if appropriate).	N/A (Methods, critical appraisal)
Summary measures	13	*Not applicable for scoping reviews*	*N/A*
Synthesis of results	14	Describe the methods of handling and summarizing the data that were charted.	Methods, data synthesis
Risk of bias across studies	15	*Not applicable for scoping reviews*	*N/A*
Additional analyses	16	*Not applicable for scoping reviews*	*N/A*
**Results**			
Selection of sources of evidence	17	Give numbers of sources of evidence screened, assessed for eligibility, and included in the review, with reasons for exclusions at each stage, ideally using a flow diagram.	Results, study selection and Figure 1, Supplementary file 3 and 4
Characteristics of sources of evidence	18	For each source of evidence, present characteristics for which data were charted and provide the citations.	Results, Tables 1-5
Critical appraisal within sources of evidence	19	If done, present data on critical appraisal of included sources of evidence (see item 12).	N/A
Results of individual sources of evidence	20	For each included source of evidence, present the relevant data that were charted that relate to the review questions and objectives.	Not shown, can be provided by the authors

Synthesis of results	21	Summarize and/or present the charting results as they relate to the review questions and objectives.	Table 6, Figures 2-5
Risk of bias across studies	22	*Not applicable for scoping reviews*	*N/A*
Additional analyses	23	*Not applicable for scoping reviews*	*N/A*
**Discussion**			
Summary of evidence	24	Summarize the main results (including an overview of concepts, themes, and types of evidence available), link to the review questions and objectives, and consider the relevance to key groups.	Discussion, summary of main findings
Limitations	25	Discuss the limitations of the scoping review process.	Discussion, limitations
Conclusions	26	Provide a general interpretation of the results with respect to the review questions and objectives, as well as potential implications and/or next steps.	Discussion, conclusions
**Funding**	27	Describe sources of funding for the included sources of evidence, as well as sources of funding for the scoping review. Describe the role of the funders of the scoping review.	Funding

Source: Tricco AC, Lillie E, Zarin W, O'Brien KK, Colquhoun H, Levac D, et al. Prisma Extension For Scoping Reviews (Prisma-Scr): Checklist and explanation. Ann Intern Med. 2018;169(7):467-73. doi: 10.7326/m18-0850.

Search strategy

Search date	ORIGINAL: 14.05.2024			
search number	search strategy	MeSH	Limit	search results
#1	MeSH descriptor: [Exercise] explode all trees	Yes	-	38714
#2	(Exercise* OR "Physical Activity" OR "Physical Activities")	No	ti,ab,kw	167135
#3	("physical inactivity")	No	ti,ab,kw	1826
#4	(exercising)	No	ti,ab,kw	3211
#5	(sedentary)	No	ti,ab,kw	10299
#6	#1 OR #2 OR #3 OR #4 OR #5 in Cochrane Reviews	-	Cochrane Reviews	836
#7	MeSH descriptor: [Diet, Food, and Nutrition] explode all trees	Yes	-	77376
#8	(diet* or food* or nutrition*)	No	ti,ab,kw	177732
#9	(Healthy AND Eating)	No	ti,ab,kw	6814
#10	#7 OR #8 OR #9 in Cochrane Reviews	-	Cochrane Reviews	1256
#11	#6 OR #10	-	Cochrane Reviews	**1912**

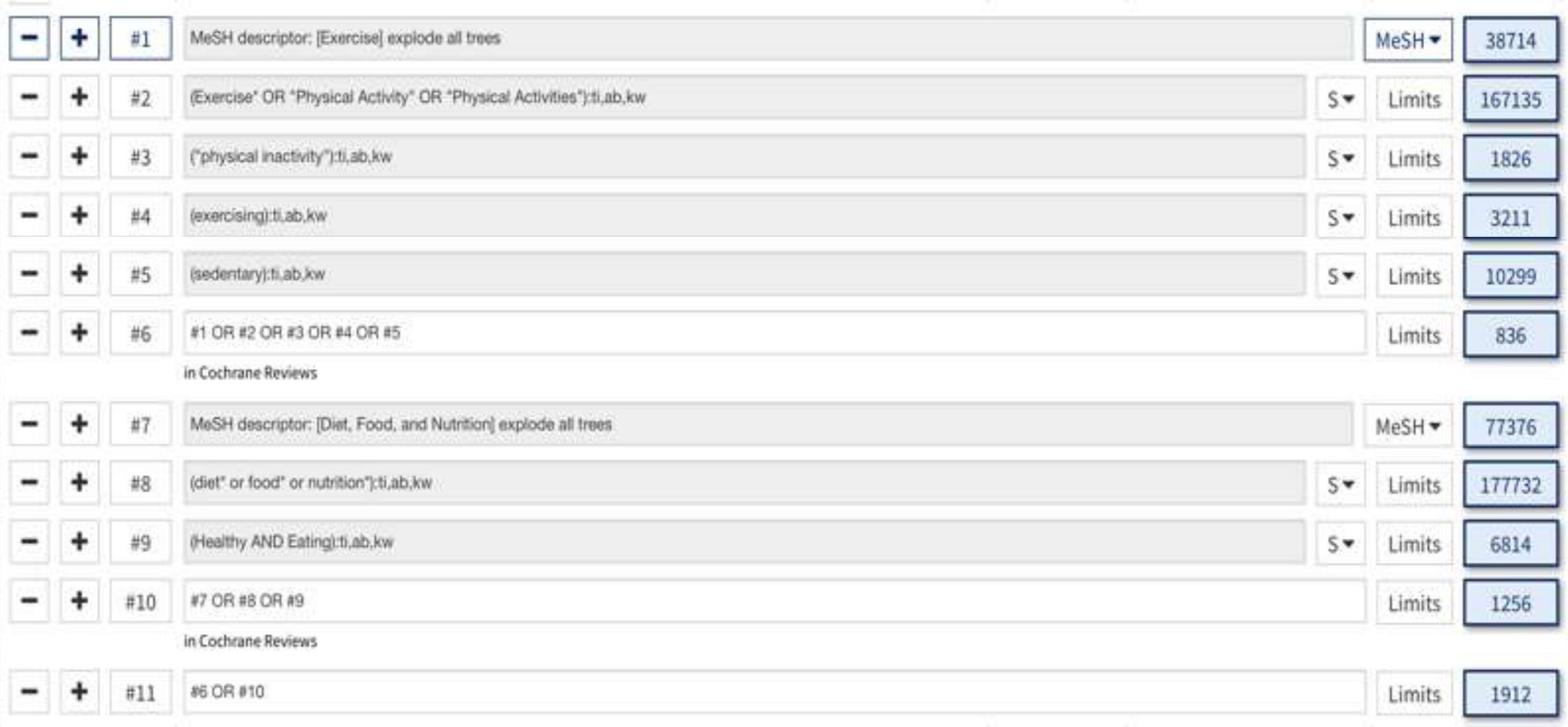

Search date	UPDATE: 22.01.2026	
search number	search strategy	search results
#1	MeSH descriptor: [Exercise] explode all trees	41880
#2	(Exercise* OR "Physical Activity" OR "Physical Activities"):ti,ab,kw	191010
#3	("physical inactivity"):ti,ab,kw	2058
#4	(exercising):ti,ab,kw	3495
#5	(sedentary):ti,ab,kw	11521
#6	#1 OR #2 OR #3 OR #4 OR #5	197611
#7	MeSH descriptor: [Diet, Food, and Nutrition] explode all trees	78108
#8	(diet* or food* or nutrition*):ti,ab,kw	193815
#9	(Healthy AND Eating):ti,ab,kw	7548
#10	#7 OR #8 OR #9	213223
#11	#6 OR #10	367687
#12	#11 (limit: published between May 2024 and Jan 2026)	131

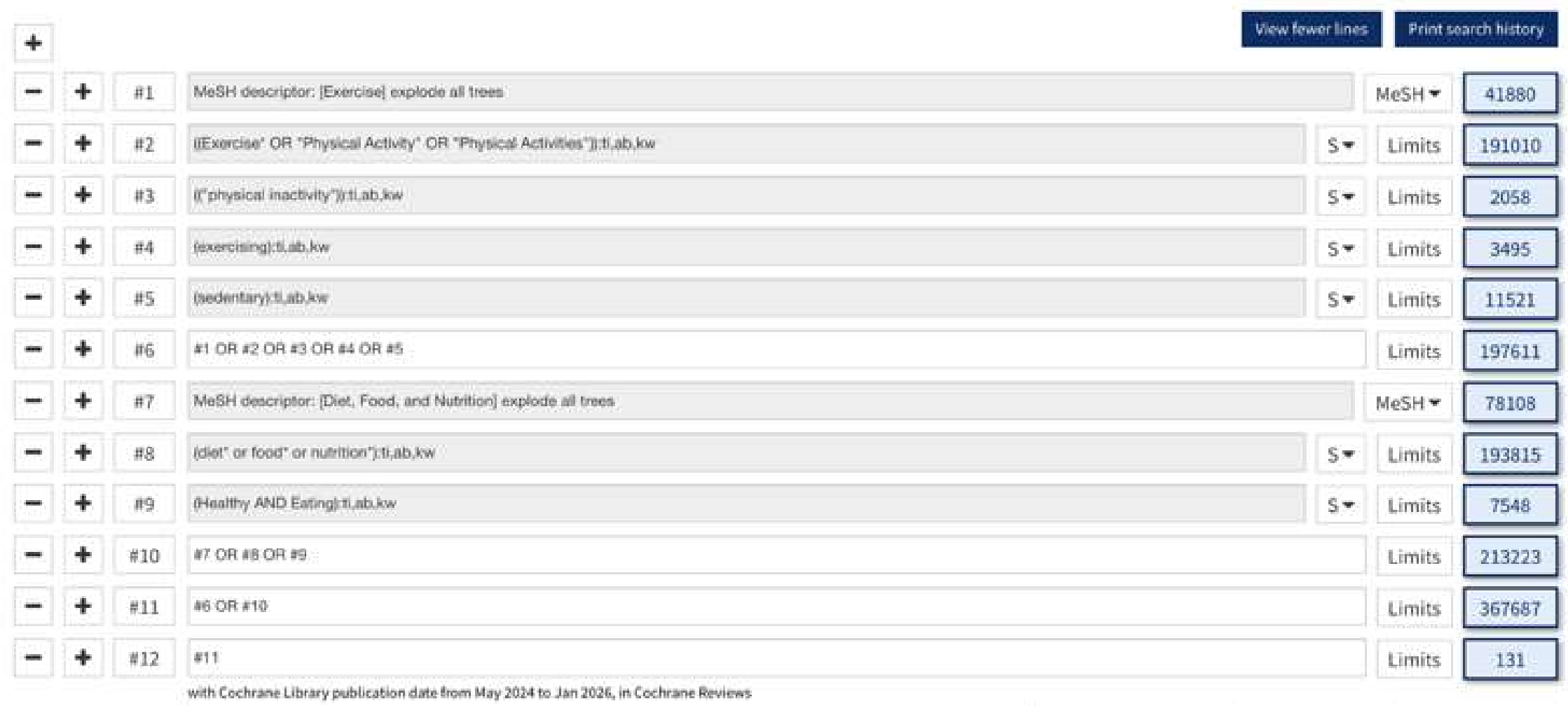
View fewer lines
Print search history

#1	MeSH descriptor: [Exercise] explode all trees	MeSH		41880
#2	((Exercise* OR "Physical Activity" OR "Physical Activities")):ti,ab,kw	S	Limits	191010
#3	(("physical inactivity")):ti,ab,kw	S	Limits	2058
#4	(exercising):ti,ab,kw	S	Limits	3495
#5	(sedentary):ti,ab,kw	S	Limits	11521
#6	#1 OR #2 OR #3 OR #4 OR #5		Limits	197611
#7	MeSH descriptor: [Diet, Food, and Nutrition] explode all trees	MeSH		78108
#8	(diet* or food* or nutrition*):ti,ab,kw	S	Limits	193815
#9	(Healthy AND Eating):ti,ab,kw	S	Limits	7548
#10	#7 OR #8 OR #9		Limits	213223
#11	#6 OR #10		Limits	367687
#12	#11		Limits	131

with Cochrane Library publication date from May 2024 to Jan 2026, in Cochrane Reviews

Excluded studies (n=66)

Excluded studies	Exclusion reason
[1-29]	Exclusion 1 (clinical populations)
[30-50]	Exclusion 2 (no primary focus on nutrition or PA)
[51-66]	Exclusion 3 (older reviews with updates already included in this review)

1. Bae, S., et al., *Provision of folic acid for reducing arsenic toxicity in arsenic-exposed children and adults.* Cochrane Database of Systematic Reviews, 2021(10).
2. Bello, S., et al., *Routine vitamin A supplementation for the prevention of blindness due to measles infection in children.* Cochrane Database of Systematic Reviews, 2016(8).
3. Cameron, I., et al., *Interventions for preventing falls in older people in care facilities and hospitals.* Cochrane Database of Systematic Reviews, 2018(9).
4. Chibuzor, M., et al., *Vitamin D, calcium or a combination of vitamin D and calcium for the treatment of nutritional rickets in children.* Cochrane Database of Systematic Reviews, 2020(4).
5. Crocker, T., et al., *Physical rehabilitation for older people in long-term care.* Cochrane Database of Systematic Reviews, 2013(2).
6. Darlow, B., P. Graham, and M. Rojas-Reyes, *Vitamin A supplementation to prevent mortality and short- and long-term morbidity in very low birth weight infants.* Cochrane Database of Systematic Reviews, 2016(8).
7. Skelton, D., et al., *Environmental and behavioural interventions for reducing physical activity limitation and preventing falls in older people with visual impairment.* Cochrane Database of Systematic Reviews, 2020(9).
8. Edwards, T., et al., *Oral dextrose gel for the treatment of hypoglycaemia in newborn infants.* Cochrane Database of Systematic Reviews, 2022(3).
9. Esmaeilinezhad, Z., et al., *Probiotics for the prevention of Clostridioides difficile-associated diarrhea in adults and children.* Cochrane Database of Systematic Reviews, 2025(9).
10. Fernandes, A., et al., *Pelvic floor muscle training with feedback or biofeedback for urinary incontinence in women.* Cochrane Database of Systematic Reviews, 2025(3).
11. Fernández-Gaxiola, A. and L. De-Regil, *Intermittent iron supplementation for reducing anaemia and its associated impairments in adolescent and adult menstruating women.* Cochrane Database of Systematic Reviews, 2019(1).
12. Flint, A., K. New, and M. Davies, *Cup feeding versus other forms of supplemental enteral feeding for newborn infants unable to fully breastfeed.* Cochrane Database of Systematic Reviews, 2016(8).
13. Kern, A., et al., *Medical and dietary interventions for preventing recurrent urinary stones in children.* Cochrane Database of Systematic Reviews, 2017(11).
14. Kleijnen, J. and D. Mackerras, *Vitamin E for intermittent claudication.* Cochrane Database of Systematic Reviews, 1998(1).
15. Langer, G., et al., *Nutritional interventions for preventing and treating pressure ulcers.* Cochrane Database of Systematic Reviews, 2024(2).
16. Malhotra, A., et al., *Dietary interventions (plant sterols, stanols, omega-3 fatty acids, soy protein and dietary fibers) for familial hypercholesterolaemia.* Cochrane Database of Systematic Reviews, 2014(6).

17. Méndez-Sánchez, L., et al., *Calcium and vitamin D for increasing bone mineral density in premenopausal women.* Cochrane Database of Systematic Reviews, 2023(1).
18. Nicolaï, S., et al., *Ginkgo biloba for intermittent claudication.* Cochrane Database of Systematic Reviews, 2013(6).
19. Palacios, C., et al., *Digital health, technology-driven or technology-assisted interventions for the management of obesity in children and adolescents.* Cochrane Database of Systematic Reviews, 2025(7).
20. Ried, K., P. Fakler, and N. Stocks, *Effect of cocoa on blood pressure.* Cochrane Database of Systematic Reviews, 2017(4).
21. Saragiotto, B., et al., *Motor control exercise for chronic non-specific low-back pain.* Cochrane Database of Systematic Reviews, 2016(1).
22. Schoonees, A., et al., *Ready-to-use therapeutic food (RUTF) for home-based nutritional rehabilitation of severe acute malnutrition in children from six months to five years of age.* Cochrane Database of Systematic Reviews, 2019(5).
23. Tan, M., J. Ho, and K. Teh, *Polyunsaturated fatty acids (PUFAs) for children with specific learning disorders.* Cochrane Database of Systematic Reviews, 2016(9).
24. Treacy, D., et al., *Mobility training for increasing mobility and functioning in older people with frailty.* Cochrane Database of Systematic Reviews, 2022(6).
25. Voinescu, A., et al., *Exergaming for dementia and mild cognitive impairment.* Cochrane Database of Systematic Reviews, 2024(9).
26. Wang, H., et al., *Oral vitamin B12 versus intramuscular vitamin B12 for vitamin B12 deficiency.* Cochrane Database of Systematic Reviews, 2018(3).
27. Wen, H., et al., *Vitamin E for people with non-alcoholic fatty liver disease.* Cochrane Database of Systematic Reviews, 2024(10).
28. Whitford, H., et al., *Breastfeeding education and support for women with twins or higher order multiples.* Cochrane Database of Systematic Reviews, 2017(2).
29. Zehetner, A., et al., *Iron supplementation for breath-holding attacks in children.* Cochrane Database of Systematic Reviews, 2010(5).
30. Clasen, T., et al., *Interventions to improve water quality for preventing diarrhoea.* Cochrane Database of Systematic Reviews, 2015(10).
31. Cooper, A., et al., *Primary school-based behavioural interventions for preventing caries.* Cochrane Database of Systematic Reviews, 2013(5).
32. Desroches, S., et al., *Interventions to enhance adherence to dietary advice for preventing and managing chronic diseases in adults.* Cochrane Database of Systematic Reviews, 2013(2).
33. Ekeland, E., et al., *Exercise to improve self-esteem in children and young people.* Cochrane Database of Systematic Reviews, 2004(1).
34. Grimley Evans, J., et al., *Dehydroepiandrosterone (DHEA) supplementation for cognitive function in healthy elderly people.* Cochrane Database of Systematic Reviews, 2006(4).
35. Hooper, L., et al., *Effects of total fat intake on body weight.* Cochrane Database of Systematic Reviews, 2015(8).
36. Hooper, L., et al., *Effects of total fat intake on body fatness in adults.* Cochrane Database of Systematic Reviews, 2020(6).
37. Husk, K., et al., *Participation in environmental enhancement and conservation activities for health and well-being in adults: a review of quantitative and qualitative evidence.* Cochrane Database of Systematic Reviews, 2016(5).

38. Jaafar, S., et al., *Effect of restricted pacifier use in breastfeeding term infants for increasing duration of breastfeeding.* Cochrane Database of Systematic Reviews, 2016(8).
39. Jaafar, S., J. Ho, and K. Lee, *Rooming-in for new mother and infant versus separate care for increasing the duration of breastfeeding.* Cochrane Database of Systematic Reviews, 2016(8).
40. Lee, H., et al., *Ginseng for erectile dysfunction.* Cochrane Database of Systematic Reviews, 2021(4).
41. McClure, R., et al., *Population-based interventions for the prevention of fall-related injuries in older people.* Cochrane Database of Systematic Reviews, 2005(1).
42. Osborn, D., et al., *Higher versus lower amino acid intake in parenteral nutrition for newborn infants.* Cochrane Database of Systematic Reviews, 2018(3).
43. Ramamurthy, P., et al., *Sealants for preventing dental caries in primary teeth.* Cochrane Database of Systematic Reviews, 2022(2).
44. Taylor, M., et al., *The impact of growth monitoring and promotion on health indicators in children under five years of age in low- and middle-income countries.* Cochrane Database of Systematic Reviews, 2023(10).
45. Whitworth, M. and T. Dowswell, *Routine pre-pregnancy health promotion for improving pregnancy outcomes.* Cochrane Database of Systematic Reviews, 2009(4).
46. Wolfenden, L., et al., *Strategies to improve the implementation of workplace-based policies or practices targeting tobacco, alcohol, diet, physical activity and obesity.* Cochrane Database of Systematic Reviews, 2018(11).
47. Fandim, J.V., et al., *Telerehabilitation for neck pain.* Cochrane Database of Systematic Reviews, 2025(8).
48. Huey, S.L., et al., *Precision nutrition-based interventions for the management of obesity in children and adolescents up to the age of 19 years.* Cochrane Database of Systematic Reviews, 2025(1).
49. Luger, T., et al., *Work-break interventions for preventing musculoskeletal symptoms and disorders in healthy workers.* Cochrane Database of Systematic Reviews, 2025(10).
50. Rizzo, R.R.N., et al., *Non-pharmacological and non-surgical treatments for low back pain in adults: an overview of Cochrane reviews.* Cochrane Database of Systematic Reviews, 2025(3).
51. Brown, T., et al., *Interventions for preventing obesity in children.* Cochrane Database of Systematic Reviews, 2019(7).
52. Buppasiri, P., et al., *Calcium supplementation (other than for preventing or treating hypertension) for improving pregnancy and infant outcomes.* Cochrane Database of Systematic Reviews, 2015(2).
53. Hodder, R.K., et al., *Interventions for increasing fruit and vegetable consumption in children aged five years and under.* Cochrane Database of Systematic Reviews, 2020(5).
54. Hofmeyr, G., et al., *Calcium supplementation during pregnancy for preventing hypertensive disorders and related problems.* Cochrane Database of Systematic Reviews, 2018(10).
55. Hofmeyr, G.J., et al., *Calcium supplementation commencing before or early in pregnancy, for preventing hypertensive disorders of pregnancy.* Cochrane Database of Systematic Reviews, 2019(9).

56. Iheozor-Ejiofor, Z., et al., *Water fluoridation for the prevention of dental caries.* Cochrane Database of Systematic Reviews, 2015(6).
57. Kramer, M., *High protein supplementation in pregnancy.* Cochrane Database of Systematic Reviews, 1996(4).
58. Kramer, M., *Maternal antigen avoidance during lactation for preventing atopic eczema in infants.* Cochrane Database of Systematic Reviews, 1996(4).
59. Kristjansson, B., et al., *School feeding for improving the physical and psychosocial health of disadvantaged students.* Cochrane Database of Systematic Reviews, 2007(1).
60. Neuberger, A., et al., *Oral iron supplements for children in malaria‐endemic areas.* Cochrane Database of Systematic Reviews, 2016(2).
61. Osborn, D. and J. Sinn, *Probiotics in infants for prevention of allergic disease and food hypersensitivity.* Cochrane Database of Systematic Reviews, 2007(4).
62. Palacios, C., L.K. Kostiuk, and J.P. Peña-Rosas, *Vitamin D supplementation for women during pregnancy.* Cochrane Database of Systematic Reviews, 2019(7).
63. Peña-Rosas, J.P., et al., *Daily oral iron supplementation during pregnancy.* Cochrane Database of Systematic Reviews, 2015(7).
64. Priest, N., et al., *Interventions implemented through sporting organisations for increasing participation in sport.* Cochrane Database of Systematic Reviews, 2008(3).
65. Riggs, E., et al., *Interventions with pregnant women, new mothers and other primary caregivers for preventing early childhood caries.* Cochrane Database of Systematic Reviews, 2019(11).
66. Wolfenden, L., et al., *Strategies for enhancing the implementation of school‐based policies or practices targeting diet, physical activity, obesity, tobacco or alcohol use.* Cochrane Database of Systematic Reviews, 2022(8).

Included studies n=260

1. Aas, R., Tuntland, H., Holte, K., Røe, C., Lund, T., Marklund, S., & Moller, A. (2011). Workplace interventions for neck pain in workers. *Cochrane Database of Systematic Reviews*(4). https://doi.org/doi:10.1002/14651858.CD008160.pub2
2. Abdelhamid, A. S., Brown, T. J., Brainard, J. S., Biswas, P., Thorpe, G. C., Moore, H. J., Deane, K. H. O., Summerbell, C. D., Worthington, H. V., Song, F., & et al. (2020). Omega-3 fatty acids for the primary and secondary prevention of cardiovascular disease. *Cochrane Database of Systematic Reviews*(3). https://doi.org/10.1002/14651858.CD003177.pub5
3. Abdelhamid, A. S., Martin, N., Bridges, C., Brainard, J. S., Wang, X., Brown, T. J., Hanson, S., Jimoh, O. F., Ajabnoor, S. M., Deane, K. H. O., & et al. (2018). Polyunsaturated fatty acids for the primary and secondary prevention of cardiovascular disease. *Cochrane Database of Systematic Reviews*(11). https://doi.org/10.1002/14651858.CD012345.pub3
4. Abdulwadud, O., & Snow, M. (2012). Interventions in the workplace to support breastfeeding for women in employment. *Cochrane Database of Systematic Reviews*(10). https://doi.org/doi:10.1002/14651858.CD006177.pub3
5. Abe, S. K., Balogun, O. O., Ota, E., Takahashi, K., & Mori, R. (2016). Supplementation with multiple micronutrients for breastfeeding women for improving outcomes for the mother and baby. *Cochrane Database of Systematic Reviews*(2). https://doi.org/10.1002/14651858.CD010647.pub2
6. Adler, A. J., Taylor, F., Martin, N., Gottlieb, S., Taylor, R. S., & Ebrahim, S. (2014). Reduced dietary salt for the prevention of cardiovascular disease. *Cochrane Database of Systematic Reviews*(12). https://doi.org/10.1002/14651858.CD009217.pub3
7. Al-Khudairy, L., Flowers, N., Wheelhouse, R., Ghannam, O., Hartley, L., Stranges, S., & Rees, K. (2017). Vitamin C supplementation for the primary prevention of cardiovascular disease. *Cochrane Database of Systematic Reviews*(3). https://doi.org/10.1002/14651858.CD011114.pub2
8. Al-Khudairy, L., Loveman, E., Colquitt, J., Mead, E., Johnson, R., Fraser, H., Olajide, J., Murphy, M., Velho, R., O'Malley, C., & et al. (2017). Diet, physical activity and behavioural interventions for the treatment of overweight or obese adolescents aged 12 to 17 years. *Cochrane Database of Systematic Reviews*(6). https://doi.org/doi:10.1002/14651858.CD012691
9. Amorim Adegboye, A. R., & Linne, Y. M. (2013). Diet or exercise, or both, for weight reduction in women after childbirth. *Cochrane Database of Systematic Reviews*(7). https://doi.org/10.1002/14651858.CD005627.pub3
10. Arikpo, D., Edet, E., Chibuzor, M., Odey, F., & Caldwell, D. (2018). Educational interventions for improving primary caregiver complementary feeding practices for children aged 24 months and under. *Cochrane Database of Systematic Reviews*(5). https://doi.org/doi:10.1002/14651858.CD011768.pub2
11. Armanian, A., Jahanfar, S., Feizi, A., Salehimehr, N., Molaeinezhad, M., & Sadeghi, E. (2019). Prebiotics for the prevention of hyperbilirubinaemia in neonates. *Cochrane Database of Systematic Reviews*(8). https://doi.org/doi:10.1002/14651858.CD012731.pub2
12. Ashworth, N. L., Chad, K. E., Harrison, E. L., Reeder, B. A., & Marshall, S. C. (2005). Home versus center based physical activity programs in older adults. *Cochrane Database of Systematic Reviews*(1). https://doi.org/10.1002/14651858.CD004017.pub2
13. Avenell, A., Mak, J. C. S., & O'Connell, D. L. (2014). Vitamin D and vitamin D analogues for preventing fractures in post-menopausal women and older men. *Cochrane Database of Systematic Reviews*(4). https://doi.org/10.1002/14651858.CD000227.pub4
14. Azarpazhooh, A., Lawrence, H., & Shah, P. (2016). Xylitol for preventing acute otitis media in children up to 12 years of age. *Cochrane Database of Systematic Reviews*(8). https://doi.org/doi:10.1002/14651858.CD007095.pub3

15. Baker, P. R. A., Francis, D. P., Soares, J., Weightman, A. L., & Foster, C. (2015). Community wide interventions for increasing physical activity. *Cochrane Database of Systematic Reviews*(1). https://doi.org/10.1002/14651858.CD008366.pub3
16. Baldwin, C., Kimber, K., Gibbs, M., & Weekes, C. (2016). Supportive interventions for enhancing dietary intake in malnourished or nutritionally at-risk adults. *Cochrane Database of Systematic Reviews*(12). https://doi.org/doi:10.1002/14651858.CD009840.pub2
17. Balogun, O., da Silva Lopes, K., Ota, E., Takemoto, Y., Rumbold, A., Takegata, M., & Mori, R. (2016). Vitamin supplementation for preventing miscarriage. *Cochrane Database of Systematic Reviews*(5). https://doi.org/doi:10.1002/14651858.CD004073.pub4
18. Balogun, O., O'Sullivan, E., McFadden, A., Ota, E., Gavine, A., Garner, C., Renfrew, M., & MacGillivray, S. (2016). Interventions for promoting the initiation of breastfeeding. *Cochrane Database of Systematic Reviews*(11). https://doi.org/doi:10.1002/14651858.CD001688.pub3
19. Baxter, J. A., Carducci, B., Kamali, M., Zlotkin, S. H., & Bhutta, Z. A. (2022). Fortification of salt with iron and iodine versus fortification of salt with iodine alone for improving iron and iodine status. *Cochrane Database of Systematic Reviews*(4). https://doi.org/10.1002/14651858.CD013463.pub2
20. Bensaaud, A., Seery, S., Gibson, I., Jones, J., Flaherty, G., McEvoy, J. W., Jordan, F., Tawfick, W., & Sultan, S. A. H. (2025). Dietary Approaches to Stop Hypertension (DASH) for the primary and secondary prevention of cardiovascular diseases. *Cochrane Database of Systematic Reviews*(5). https://doi.org/10.1002/14651858.CD013729.pub2
21. Bergwall, S., Johansson, A., Sonestedt, E., & Acosta, S. (2022). High versus low-added sugar consumption for the primary prevention of cardiovascular disease. *Cochrane Database of Systematic Reviews*(1). https://doi.org/10.1002/14651858.CD013320.pub2
22. Bjelakovic, G., Gluud, L. L., Nikolova, D., Whitfield, K., Krstic, G., Wetterslev, J., & Gluud, C. (2014). Vitamin D supplementation for prevention of cancer in adults. *Cochrane Database of Systematic Reviews*(6). https://doi.org/10.1002/14651858.CD007469.pub2
23. Bjelakovic, G., Gluud, L. L., Nikolova, D., Whitfield, K., Wetterslev, J., Simonetti, R. G., Bjelakovic, M., & Gluud, C. (2014). Vitamin D supplementation for prevention of mortality in adults. *Cochrane Database of Systematic Reviews*(1). https://doi.org/10.1002/14651858.CD007470.pub3
24. Bjelakovic, G., Nikolova, D., Simonetti, R. G., & Gluud, C. (2008). Antioxidant supplements for preventing gastrointestinal cancers. *Cochrane Database of Systematic Reviews*(3). https://doi.org/10.1002/14651858.CD004183.pub3
25. Bradbury, K. E., Coffey, S., Earle, N., Ni Mhurchu, C., & Jull, A. B. (2025). Riboflavin supplements for blood pressure lowering in adults. *Cochrane Database of Systematic Reviews*(10). https://doi.org/10.1002/14651858.CD015464.pub2
26. Brand, A., Visser, M. E., Schoonees, A., & Naude, C. E. (2022). Replacing salt with low-sodium salt substitutes (LSSS) for cardiovascular health in adults, children and pregnant women. *Cochrane Database of Systematic Reviews*(8). https://doi.org/10.1002/14651858.CD015207
27. Bricker, L., Reed, K., Wood, L., & Neilson, J. P. (2015). Nutritional advice for improving outcomes in multiple pregnancies. *Cochrane Database of Systematic Reviews*(11). https://doi.org/10.1002/14651858.CD008867.pub3
28. Carducci, B., Keats, E., & Bhutta, Z. (2021). Zinc supplementation for improving pregnancy and infant outcome. *Cochrane Database of Systematic Reviews*(3). https://doi.org/doi:10.1002/14651858.CD000230.pub6
29. Centeno Tablante, E., Pachón, H., Guetterman, H. M., & Finkelstein, J. L. (2019). Fortification of wheat and maize flour with folic acid for population health outcomes. *Cochrane Database of Systematic Reviews*(7). https://doi.org/10.1002/14651858.CD012150.pub2
30. Chastin, S., Gardiner, P. A., Harvey, J. A., Leask, C. F., Jerez-Roig, J., Rosenberg, D., Ashe, M. C., Helbostad, J. L., & Skelton, D. A. (2021). Interventions for reducing sedentary behaviour in community-dwelling older adults. *Cochrane Database of Systematic Reviews*(6). https://doi.org/10.1002/14651858.CD012784.pub2

31. Chekima, K., Yan, S., Lee, S. H., Wong, T., Noor, M., Ooi, Y., Metzendorf, M.-I., & Lai, N. (2023). Low glycaemic index or low glycaemic load diets for people with overweight or obesity. *Cochrane Database of Systematic Reviews*(6). https://doi.org/doi:10.1002/14651858.CD005105.pub3
32. Chen, H., Zhuo, Q., Yuan, W., Wang, J., & Wu, T. (2008). Vitamin A for preventing acute lower respiratory tract infections in children up to seven years of age. *Cochrane Database of Systematic Reviews*(1). https://doi.org/doi:10.1002/14651858.CD006090.pub2
33. Chen, N., Yang, M., Zhou, M., Xiao, J., Guo, J., & He, L. (2017). L-carnitine for cognitive enhancement in people without cognitive impairment. *Cochrane Database of Systematic Reviews*(3). https://doi.org/10.1002/14651858.CD009374.pub3
34. Cheng, X., Li, D., Yang, C., Chen, B., Xu, P., & Zhang, L. (2024). Oral vitamin A supplements to prevent acute upper respiratory tract infections in children up to seven years of age. *Cochrane Database of Systematic Reviews*(5). https://doi.org/10.1002/14651858.CD015306.pub2
35. Choi, B. K. L., Verbeek, J. H., Tam, W. W. S., & Jiang, J. Y. (2010). Exercises for prevention of recurrences of low-back pain. *Cochrane Database of Systematic Reviews*(1). https://doi.org/10.1002/14651858.CD006555.pub2
36. Clar, C., Al-Khudairy, L., Loveman, E., Kelly, S. A. M., Hartley, L., Flowers, N., Germanò, R., Frost, G., & Rees, K. (2017). Low glycaemic index diets for the prevention of cardiovascular disease. *Cochrane Database of Systematic Reviews*(7). https://doi.org/10.1002/14651858.CD004467.pub3
37. Clarke, N., Pechey, E., Shemilt, I., Pilling, M., Roberts, N. W., Marteau, T. M., Jebb, S. A., & Hollands, G. J. (2025). Calorie (energy) labelling for changing selection and consumption of food or alcohol. *Cochrane Database of Systematic Reviews*(1). https://doi.org/10.1002/14651858.CD014845.pub2
38. Cluver, C. A., Rohwer, C., Rohwer, A., Puga, M., Torloni, M. R., & Hofmeyr, G. J. (2025). Calcium supplementation commenced before pregnancy for preventing hypertensive disorders and related problems. *Cochrane Database of Systematic Reviews*(9). https://doi.org/10.1002/14651858.CD011192.pub4
39. Cluver, C. A., Rohwer, C., & Rohwer, A. C. (2025). Calcium supplementation during pregnancy for preventing hypertensive disorders and related problems. *Cochrane Database of Systematic Reviews*(12). https://doi.org/10.1002/14651858.CD001059.pub6
40. Colquitt, J., Loveman, E., O'Malley, C., Azevedo, L., Mead, E., Al-Khudairy, L., Ells, L., Metzendorf, M., & Rees, K. (2016). Diet, physical activity, and behavioural interventions for the treatment of overweight or obesity in preschool children up to the age of 6 years. *Cochrane Database of Systematic Reviews*(3). https://doi.org/doi:10.1002/14651858.CD012105
41. Cooper, T., Teng, C., Howell, M., Teixeira-Pinto, A., Jaure, A., & Wong, G. (2022). D-mannose for preventing and treating urinary tract infections. *Cochrane Database of Systematic Reviews*(8). https://doi.org/doi:10.1002/14651858.CD013608.pub2
42. Cormick, G., Ciapponi, A., Cafferata, M. L., Cormick, M. S., & Belizán, J. M. (2022). Calcium supplementation for prevention of primary hypertension. *Cochrane Database of Systematic Reviews*(1). https://doi.org/10.1002/14651858.CD010037.pub4
43. Cormick, G., Ciapponi, A., Harbron, J., Perez, S. M., Vazquez, P., Rivo, J., Metzendorf, M. I., Althabe, F., & Belizán, J. M. (2024). Calcium supplementation for people with overweight or obesity. *Cochrane Database of Systematic Reviews*(5). https://doi.org/10.1002/14651858.CD012268.pub2
44. Crockett, R. A., King, S. E., Marteau, T. M., Prevost, A. T., Bignardi, G., Roberts, N. W., Stubbs, B., Hollands, G. J., & Jebb, S. A. (2018). Nutritional labelling for healthier food or non-alcoholic drink purchasing and consumption. *Cochrane Database of Systematic Reviews*(2). https://doi.org/10.1002/14651858.CD009315.pub2
45. da Silva Lopes, K., Yamaji, N., Rahman, M., Suto, M., Takemoto, Y., Garcia-Casal, M., & Ota, E. (2021). Nutrition-specific interventions for preventing and controlling anaemia throughout the life cycle: an overview of systematic reviews. *Cochrane Database of Systematic Reviews*(9). https://doi.org/doi:10.1002/14651858.CD013092.pub2

46. Dangour, A. D., Watson, L., Cumming, O., Boisson, S., Che, Y., Velleman, Y., Cavill, S., Allen, E., & Uauy, R. (2013). Interventions to improve water quality and supply, sanitation and hygiene practices, and their effects on the nutritional status of children. *Cochrane Database of Systematic Reviews*(8). https://doi.org/10.1002/14651858.CD009382.pub2
47. Das, J. K., Hoodbhoy, Z., Salam, R. A., Bhutta, A. Z., Valenzuela-Rubio, N. G., Weise Prinzo, Z., & Bhutta, Z. A. (2018). Lipid-based nutrient supplements for maternal, birth, and infant developmental outcomes. *Cochrane Database of Systematic Reviews*(8). https://doi.org/10.1002/14651858.CD012610.pub2
48. Das, J. K., Salam, R. A., Hadi, Y. B., Sadiq Sheikh, S., Bhutta, A. Z., Weise Prinzo, Z., & Bhutta, Z. A. (2019). Preventive lipid-based nutrient supplements given with complementary foods to infants and young children 6 to 23 months of age for health, nutrition, and developmental outcomes. *Cochrane Database of Systematic Reviews*(5). https://doi.org/10.1002/14651858.CD012611.pub3
49. Das, J. K., Salam, R. A., Mahmood, S. B., Moin, A., Kumar, R., Mukhtar, K., Lassi, Z. S., & Bhutta, Z. A. (2019). Food fortification with multiple micronutrients: impact on health outcomes in general population. *Cochrane Database of Systematic Reviews*(12). https://doi.org/10.1002/14651858.CD011400.pub2
50. Davidson, S. J., Barrett, H. L., Price, S. A., Callaway, L. K., & Dekker Nitert, M. (2021). Probiotics for preventing gestational diabetes. *Cochrane Database of Systematic Reviews*(4). https://doi.org/10.1002/14651858.CD009951.pub3
51. De-Regil, L. M., Jefferds, M. E. D., & Peña-Rosas, J. P. (2017). Point-of-use fortification of foods with micronutrient powders containing iron in children of preschool and school-age. *Cochrane Database of Systematic Reviews*(11). https://doi.org/10.1002/14651858.CD009666.pub2
52. De-Regil, L. M., Jefferds, M. E. D., Sylvetsky, A. C., & Dowswell, T. (2011). Intermittent iron supplementation for improving nutrition and development in children under 12 years of age. *Cochrane Database of Systematic Reviews*(12). https://doi.org/10.1002/14651858.CD009085.pub2
53. De-Regil, L. M., Peña-Rosas, J. P., Fernández-Gaxiola, A. C., & Rayco-Solon, P. (2015). Effects and safety of periconceptional oral folate supplementation for preventing birth defects. *Cochrane Database of Systematic Reviews*(12). https://doi.org/10.1002/14651858.CD007950.pub3
54. Delgado-Noguera, M. F., Calvache, J. A., Bonfill Cosp, X., Kotanidou, E. P., & Galli-Tsinopoulou, A. (2015). Supplementation with long chain polyunsaturated fatty acids (LCPUFA) to breastfeeding mothers for improving child growth and development. *Cochrane Database of Systematic Reviews*(7). https://doi.org/10.1002/14651858.CD007901.pub3
55. Dickinson, H., Bain, E., Wilkinson, D., Middleton, P., Crowther, C., & Walker, D. (2014). Creatine for women in pregnancy for neuroprotection of the fetus. *Cochrane Database of Systematic Reviews*(12). https://doi.org/doi:10.1002/14651858.CD010846.pub2
56. Duley, L., Henderson-Smart, D., & Meher, S. (2005). Altered dietary salt for preventing pre-eclampsia, and its complications. *Cochrane Database of Systematic Reviews*(4). https://doi.org/doi:10.1002/14651858.CD005548
57. Duley, L., & Henderson-Smart, D. J. (1999). Reduced salt intake compared to normal dietary salt, or high intake, in pregnancy. *Cochrane Database of Systematic Reviews*(3). https://doi.org/10.1002/14651858.CD001687
58. Durao, S., Visser, M. E., Ramokolo, V., Oliveira, J. M., Schmidt, B. M., Balakrishna, Y., Brand, A., Kristjansson, E., & Schoonees, A. (2020). Community-level interventions for improving access to food in low- and middle-income countries. *Cochrane Database of Systematic Reviews*(8). https://doi.org/10.1002/14651858.CD011504.pub3
59. Dyer, S. M., Kwok, W. S., Suen, J., Dawson, R., Kneale, D., Sutcliffe, K., Seppala, L. J., Hill, K. D., Kerse, N., Murray, G. R., & et al. (2025). Interventions for preventing falls in older people in care facilities. *Cochrane Database of Systematic Reviews*(8). https://doi.org/10.1002/14651858.CD016064
60. Eaton, J. C., Rothpletz-Puglia, P., Dreker, M. R., Iannotti, L., Lutter, C., Kaganda, J., & Rayco-Solon, P. (2019). Effectiveness of provision of animal-source foods for supporting optimal growth and development in

children 6 to 59 months of age. *Cochrane Database of Systematic Reviews*(2). https://doi.org/10.1002/14651858.CD012818.pub2

61. Ebrahim, S., Taylor, F., Ward, K., Beswick, A., Burke, M., & Davey Smith, G. (2011). Multiple risk factor interventions for primary prevention of coronary heart disease. *Cochrane Database of Systematic Reviews*(1). https://doi.org/doi:10.1002/14651858.CD001561.pub3
62. Evans, J., & Lawrenson, J. (2017). Antioxidant vitamin and mineral supplements for preventing age-related macular degeneration. *Cochrane Database of Systematic Reviews*(7). https://doi.org/doi:10.1002/14651858.CD000253.pub4
63. Fair, F. J., Ford, G. L., & Soltani, H. (2019). Interventions for supporting the initiation and continuation of breastfeeding among women who are overweight or obese. *Cochrane Database of Systematic Reviews*(9). https://doi.org/10.1002/14651858.CD012099.pub2
64. Fallon, A., Van der Putten, D., Dring, C., Moylett, E. H., Fealy, G., & Devane, D. (2016). Baby-led compared with scheduled (or mixed) breastfeeding for successful breastfeeding. *Cochrane Database of Systematic Reviews*(9). https://doi.org/10.1002/14651858.CD009067.pub3
65. Field, M., Mithra, P., & Peña-Rosas, J. (2021). Wheat flour fortification with iron and other micronutrients for reducing anaemia and improving iron status in populations. *Cochrane Database of Systematic Reviews*(1). https://doi.org/doi:10.1002/14651858.CD011302.pub3
66. Filippini, T., Malavolti, M., Borrelli, F., Izzo, A. A., Fairweather-Tait, S. J., Horneber, M., & Vinceti, M. (2020). Green tea (Camellia sinensis) for the prevention of cancer. *Cochrane Database of Systematic Reviews*(3). https://doi.org/10.1002/14651858.CD005004.pub3
67. Finkelstein, J. L., Cuthbert, A., Weeks, J., Venkatramanan, S., Larvie, D. Y., De-Regil, L. M., & Garcia-Casal, M. N. (2024). Daily oral iron supplementation during pregnancy. *Cochrane Database of Systematic Reviews*(8). https://doi.org/10.1002/14651858.CD004736.pub6
68. Finkelstein, J. L., Fothergill, A., Venkatramanan, S., Layden, A. J., Williams, J. L., Crider, K. S., & Qi, Y. P. (2024). Vitamin B12 supplementation during pregnancy for maternal and child health outcomes. *Cochrane Database of Systematic Reviews*(1). https://doi.org/10.1002/14651858.CD013823.pub2
69. Flodgren, G., Gonçalves-Bradley, D., & Summerbell, C. (2017). Interventions to change the behaviour of health professionals and the organisation of care to promote weight reduction in children and adults with overweight or obesity. *Cochrane Database of Systematic Reviews*(11). https://doi.org/doi:10.1002/14651858.CD000984.pub3
70. Flowers, N., Hartley, L., Todkill, D., Stranges, S., & Rees, K. (2014). Co-enzyme Q10 supplementation for the primary prevention of cardiovascular disease. *Cochrane Database of Systematic Reviews*(12). https://doi.org/10.1002/14651858.CD010405.pub2
71. Foong, S., Tan, M., Foong, W., Marasco, L., Ho, J., & Ong, J. (2020). Oral galactagogues (natural therapies or drugs) for increasing breast milk production in mothers of non-hospitalised term infants. *Cochrane Database of Systematic Reviews*(5). https://doi.org/doi:10.1002/14651858.CD011505.pub2
72. Foster, C., Hillsdon, M., Thorogood, M., Kaur, A., & Wedatilake, T. (2005). Interventions for promoting physical activity. *Cochrane Database of Systematic Reviews*(1). https://doi.org/10.1002/14651858.CD003180.pub2
73. Foster, C., Richards, J., Thorogood, M., & Hillsdon, M. (2013). Remote and web 2.0 interventions for promoting physical activity. *Cochrane Database of Systematic Reviews*(9). https://doi.org/10.1002/14651858.CD010395.pub2
74. Franco, J. V. A., Guo, Y., Bongaerts, B., Metzendorf, M. I., Hindemit, J., Aqra, Z., Alhalahla, M., Tapinova, K., Villegas Arbelaez, E., Alade, O. T., & et al. (2025). Multimodal health behaviour-changing interventions for children living with obesity and their parents. *Cochrane Database of Systematic Reviews*(12). https://doi.org/10.1002/14651858.CD016063
75. Franco, J. V. A., Guo, Y., Bongaerts, B., Metzendorf, M. I., Hindemit, J., Aqra, Z., Alhalahla, M., Tapinova, K., Villegas Arbelaez, E., Alade, O. T., & et al. (2025). Multimodal health behaviour-changing interventions for

adolescents living with obesity. *Cochrane Database of Systematic Reviews*(12). https://doi.org/10.1002/14651858.CD016062
76. Freak-Poli, R. L. A., Cumpston, M., Albarqouni, L., Clemes, S. A., & Peeters, A. (2020). Workplace pedometer interventions for increasing physical activity. *Cochrane Database of Systematic Reviews*(7). https://doi.org/10.1002/14651858.CD009209.pub3
77. Furber, C. M., McGowan, L., Bower, P., Kontopantelis, E., Quenby, S., & Lavender, T. (2013). Antenatal interventions for reducing weight in obese women for improving pregnancy outcome. *Cochrane Database of Systematic Reviews*(1). https://doi.org/10.1002/14651858.CD009334.pub2
78. Garcia-Casal, M., Peña-Rosas, J., De-Regil, L., Gwirtz, J., & Pasricha, S. (2018). Fortification of maize flour with iron for controlling anaemia and iron deficiency in populations. *Cochrane Database of Systematic Reviews*(12). https://doi.org/doi:10.1002/14651858.CD010187.pub2
79. Gavine, A., Shinwell, S., Buchanan, P., Farre, A., Wade, A., Lynn, F., Marshall, J., Cumming, S., Dare, S., & McFadden, A. (2022). Support for healthy breastfeeding mothers with healthy term babies. *Cochrane Database of Systematic Reviews*(10). https://doi.org/doi:10.1002/14651858.CD001141.pub6
80. Gillespie, L., Robertson, M., Gillespie, W., Sherrington, C., Gates, S., Clemson, L., & Lamb, S. (2012). Interventions for preventing falls in older people living in the community. *Cochrane Database of Systematic Reviews*(9). https://doi.org/doi:10.1002/14651858.CD007146.pub3
81. Gogia, S., & Sachdev, H. S. (2012). Zinc supplementation for mental and motor development in children. *Cochrane Database of Systematic Reviews*(12). https://doi.org/10.1002/14651858.CD007991.pub2
82. Goldenberg, J., Yap, C., Lytvyn, L., Lo, C., Beardsley, J., Mertz, D., & Johnston, B. (2017). Probiotics for the prevention of Clostridium difficile-associated diarrhea in adults and children. *Cochrane Database of Systematic Reviews*(12). https://doi.org/doi:10.1002/14651858.CD006095.pub4
83. Gomersall, J. C., Slack-Smith, L., Kilpatrick, N., Muthu, M. S., & Riggs, E. (2024). Interventions with pregnant women, new mothers and other primary caregivers for preventing early childhood caries. *Cochrane Database of Systematic Reviews*(5). https://doi.org/10.1002/14651858.CD012155.pub3
84. Gonzalez-Garay, A. G., Serralde-Zúñiga, A. E., Medina Vera, I., Velasco Hidalgo, L., & Alonso Ocaña, M. V. (2023). Higher versus lower protein intake in formula-fed term infants. *Cochrane Database of Systematic Reviews*(11). https://doi.org/10.1002/14651858.CD013758.pub2
85. Goudet, S., Bogin, B., Madise, N., & Griffiths, P. (2019). Nutritional interventions for preventing stunting in children (birth to 59 months) living in urban slums in low- and middle-income countries (LMIC). *Cochrane Database of Systematic Reviews*(6). https://doi.org/doi:10.1002/14651858.CD011695.pub2
86. Grande, A., Keogh, J., Silva, V., & Scott, A. (2020). Exercise versus no exercise for the occurrence, severity, and duration of acute respiratory infections. *Cochrane Database of Systematic Reviews*(4). https://doi.org/doi:10.1002/14651858.CD010596.pub3
87. Graudal, N. A., Hubeck-Graudal, T., & Jurgens, G. (2020). Effects of low sodium diet versus high sodium diet on blood pressure, renin, aldosterone, catecholamines, cholesterol, and triglyceride. *Cochrane Database of Systematic Reviews*(12). https://doi.org/10.1002/14651858.CD004022.pub5
88. Griffith, R. J., Alsweiler, J., Moore, A. E., Brown, S., Middleton, P., Shepherd, E., & Crowther, C. A. (2020). Interventions to prevent women from developing gestational diabetes mellitus: an overview of Cochrane Reviews. *Cochrane Database of Systematic Reviews*(6). https://doi.org/10.1002/14651858.CD012394.pub3
89. Gulani, A., & Sachdev, H. S. (2014). Zinc supplements for preventing otitis media. *Cochrane Database of Systematic Reviews*(6). https://doi.org/10.1002/14651858.CD006639.pub4
90. Gunaratne, A. W., Makrides, M., & Collins, C. T. (2015). Maternal prenatal and/or postnatal n-3 long chain polyunsaturated fatty acids (LCPUFA) supplementation for preventing allergies in early childhood. *Cochrane Database of Systematic Reviews*(7). https://doi.org/10.1002/14651858.CD010085.pub2
91. Haider, B. A., Sharma, R., & Bhutta, Z. A. (2017). Neonatal vitamin A supplementation for the prevention of mortality and morbidity in term neonates in low and middle income countries. *Cochrane Database of Systematic Reviews*(2). https://doi.org/10.1002/14651858.CD006980.pub3

92. Han, S., Middleton, P., & Crowther, C. A. (2012). Exercise for pregnant women for preventing gestational diabetes mellitus. *Cochrane Database of Systematic Reviews*(7). https://doi.org/10.1002/14651858.CD009021.pub2
93. Harding, K. B., Peña-Rosas, J. P., Webster, A. C., Yap, C. M. Y., Payne, B. A., Ota, E., & De-Regil, L. M. (2017). Iodine supplementation for women during the preconception, pregnancy and postpartum period. *Cochrane Database of Systematic Reviews*(3). https://doi.org/10.1002/14651858.CD011761.pub2
94. Harris, R., Gamboa, A., Dailey, Y., & Ashcroft, A. (2012). One-to-one dietary interventions undertaken in a dental setting to change dietary behaviour. *Cochrane Database of Systematic Reviews*(3). https://doi.org/10.1002/14651858.CD006540.pub2
95. Hartley, L., Clar, C., Ghannam, O., Flowers, N., Stranges, S., & Rees, K. (2015). Vitamin K for the primary prevention of cardiovascular disease. *Cochrane Database of Systematic Reviews*(9). https://doi.org/10.1002/14651858.CD011148.pub2
96. Hartley, L., Dyakova, M., Holmes, J., Clarke, A., Lee, M. S., Ernst, E., & Rees, K. (2014). Yoga for the primary prevention of cardiovascular disease. *Cochrane Database of Systematic Reviews*(5). https://doi.org/10.1002/14651858.CD010072.pub2
97. Hartley, L., Flowers, N., Holmes, J., Clarke, A., Stranges, S., Hooper, L., & Rees, K. (2013). Green and black tea for the primary prevention of cardiovascular disease. *Cochrane Database of Systematic Reviews*(6). https://doi.org/10.1002/14651858.CD009934.pub2
98. Hartley, L., Flowers, N., Lee, M. S., Ernst, E., & Rees, K. (2014). Tai chi for primary prevention of cardiovascular disease. *Cochrane Database of Systematic Reviews*(4). https://doi.org/10.1002/14651858.CD010366.pub2
99. Hartley, L., Igbinedion, E., Holmes, J., Flowers, N., Thorogood, M., Clarke, A., Stranges, S., Hooper, L., & Rees, K. (2013). Increased consumption of fruit and vegetables for the primary prevention of cardiovascular diseases. *Cochrane Database of Systematic Reviews*(6). https://doi.org/10.1002/14651858.CD009874.pub2
100. Hartley, L., Lee, M. S., Kwong, J. S. W., Flowers, N., Todkill, D., Ernst, E., & Rees, K. (2015). Qigong for the primary prevention of cardiovascular disease. *Cochrane Database of Systematic Reviews*(6). https://doi.org/10.1002/14651858.CD010390.pub2
101. Hartley, L., May, M. D., Loveman, E., Colquitt, J. L., & Rees, K. (2016). Dietary fibre for the primary prevention of cardiovascular disease. *Cochrane Database of Systematic Reviews*(1). https://doi.org/10.1002/14651858.CD011472.pub2
102. He, F., Li, J., & MacGregor, G. (2013). Effect of longer-term modest salt reduction on blood pressure. *Cochrane Database of Systematic Reviews*(4). https://doi.org/doi:10.1002/14651858.CD004937.pub2
103. Hemilä, H., & Chalker, E. (2013). Vitamin C for preventing and treating the common cold. *Cochrane Database of Systematic Reviews*(1). https://doi.org/10.1002/14651858.CD000980.pub4
104. Hemilä, H., & Koivula, T. (2013). Vitamin C for preventing and treating tetanus. *Cochrane Database of Systematic Reviews*(11). https://doi.org/10.1002/14651858.CD006665.pub3
105. Hemilä, H., & Louhiala, P. (2013). Vitamin C for preventing and treating pneumonia. *Cochrane Database of Systematic Reviews*(8). https://doi.org/doi:10.1002/14651858.CD005532.pub3
106. Hemmingsen, B., Gimenez-Perez, G., Mauricio, D., Roqué i Figuls, M., Metzendorf, M. I., & Richter, B. (2017). Diet, physical activity or both for prevention or delay of type 2 diabetes mellitus and its associated complications in people at increased risk of developing type 2 diabetes mellitus. *Cochrane Database of Systematic Reviews*(12). https://doi.org/10.1002/14651858.CD003054.pub4
107. Hodder, R. K., O'Brien, K. M., Al-Gobari, M., Flatz, A., Borchard, A., Klerings, I., Clinton-McHarg, T., Kingsland, M., & von Elm, E. (2025). Interventions implemented through sporting organisations for promoting healthy behaviour or improving health outcomes. *Cochrane Database of Systematic Reviews*(1). https://doi.org/10.1002/14651858.CD012170.pub2

108. Hodder, R. K., O'Brien, K. M., Wyse, R. J., Tzelepis, F., Yoong, S., Stacey, F. G., & Wolfenden, L. (2024). Interventions for increasing fruit and vegetable consumption in children aged five years and under. *Cochrane Database of Systematic Reviews*(9). https://doi.org/10.1002/14651858.CD008552.pub8
109. Hollands, G. J., Carter, P., Anwer, S., King, S. E., Jebb, S. A., Ogilvie, D., Shemilt, I., Higgins, J. P. T., & Marteau, T. M. (2019). Altering the availability or proximity of food, alcohol, and tobacco products to change their selection and consumption. *Cochrane Database of Systematic Reviews*(9). https://doi.org/10.1002/14651858.CD012573.pub3
110. Hollands, G. J., Shemilt, I., Marteau, T. M., Jebb, S. A., Lewis, H. B., Wei, Y., Higgins, J. P. T., & Ogilvie, D. (2015). Portion, package or tableware size for changing selection and consumption of food, alcohol and tobacco. *Cochrane Database of Systematic Reviews*(9). https://doi.org/10.1002/14651858.CD011045.pub2
111. Hombali, A., Solon, J., Venkatesh, B., Nair, N., & Peña-Rosas, J. (2019). Fortification of staple foods with vitamin A for vitamin A deficiency. *Cochrane Database of Systematic Reviews*(5). https://doi.org/doi:10.1002/14651858.CD010068.pub2
112. Hooper, L., Al-Khudairy, L., Abdelhamid, A. S., Rees, K., Brainard, J. S., Brown, T. J., Ajabnoor, S. M., O'Brien, A. T., Winstanley, L. E., Donaldson, D. H., & et al. (2018). Omega-6 fats for the primary and secondary prevention of cardiovascular disease. *Cochrane Database of Systematic Reviews*(11). https://doi.org/10.1002/14651858.CD011094.pub4
113. Hooper, L., Bartlett, C., Davey Smith, G., & Ebrahim, S. (2004). Advice to reduce dietary salt for prevention of cardiovascular disease. *Cochrane Database of Systematic Reviews*(1). https://doi.org/10.1002/14651858.CD003656.pub2
114. Hooper, L., Martin, N., Jimoh, O. F., Kirk, C., Foster, E., & Abdelhamid, A. S. (2020). Reduction in saturated fat intake for cardiovascular disease. *Cochrane Database of Systematic Reviews*(8). https://doi.org/10.1002/14651858.CD011737.pub3
115. Hooper, L., Summerbell, C. D., Thompson, R., Sills, D., Roberts, F. G., Moore, H. J., & Davey Smith, G. (2012). Reduced or modified dietary fat for preventing cardiovascular disease. *Cochrane Database of Systematic Reviews*(5). https://doi.org/10.1002/14651858.CD002137.pub3
116. Howe, T., Shea, B., Dawson, L., Downie, F., Murray, A., Ross, C., Harbour, R., Caldwell, L., & Creed, G. (2011). Exercise for preventing and treating osteoporosis in postmenopausal women. *Cochrane Database of Systematic Reviews*(7). https://doi.org/doi:10.1002/14651858.CD000333.pub2
117. Howe, T. E., Rochester, L., Neil, F., Skelton, D. A., & Ballinger, C. (2011). Exercise for improving balance in older people. *Cochrane Database of Systematic Reviews*(11). https://doi.org/10.1002/14651858.CD004963.pub3
118. Huey, S. L., Acharya, N., Silver, A., Sheni, R., Yu, E. A., Peña-Rosas, J. P., & Mehta, S. (2020). Effects of oral vitamin D supplementation on linear growth and other health outcomes among children under five years of age. *Cochrane Database of Systematic Reviews*(12). https://doi.org/10.1002/14651858.CD012875.pub2
119. Iheozor-Ejiofor, Z., Walsh, T., Lewis, S. R., Riley, P., Boyers, D., Clarkson, J. E., Worthington, H. V., Glenny, A. M., & O'Malley, L. (2024). Water fluoridation for the prevention of dental caries. *Cochrane Database of Systematic Reviews*(10). https://doi.org/10.1002/14651858.CD010856.pub3
120. Imdad, A., Ahmed, Z., & Bhutta, Z. A. (2016). Vitamin A supplementation for the prevention of morbidity and mortality in infants one to six months of age. *Cochrane Database of Systematic Reviews*(9). https://doi.org/10.1002/14651858.CD007480.pub3
121. Imdad, A., Mayo-Wilson, E., Haykal, M. R., Regan, A., Sidhu, J., Smith, A., & Bhutta, Z. A. (2022). Vitamin A supplementation for preventing morbidity and mortality in children from six months to five years of age. *Cochrane Database of Systematic Reviews*(3). https://doi.org/10.1002/14651858.CD008524.pub4
122. Imdad, A., Rogner, J., Sherwani, R., Sidhu, J., Regan, A., Haykal, M., Tsistinas, O., Smith, A., Chan, X., Mayo-Wilson, E., & et al. (2023). Zinc supplementation for preventing mortality, morbidity, and growth

failure in children aged 6 months to 12 years. *Cochrane Database of Systematic Reviews*(3). https://doi.org/doi:10.1002/14651858.CD009384.pub3
123. Itzkovich, M., Neuberger, A., Harris, I., Yahav, D., Paul, M., & Gilboa, M. (2026). Oral iron supplements for children in malaria-endemic areas. *Cochrane Database of Systematic Reviews*(1). https://doi.org/10.1002/14651858.CD006589.pub5
124. Jahanfar, S., & Jaafar, S. H. (2015). Effects of restricted caffeine intake by mother on fetal, neonatal and pregnancy outcomes. *Cochrane Database of Systematic Reviews*(6). https://doi.org/10.1002/14651858.CD006965.pub4
125. Jalal, C. S. B., De-Regil, L. M., Pike, V., & Mithra, P. (2023). Fortification of condiments and seasonings with iron for preventing anaemia and improving health. *Cochrane Database of Systematic Reviews*(9). https://doi.org/10.1002/14651858.CD009604.pub2
126. Jasani, B., Simmer, K., Patole, S., & Rao, S. (2017). Long chain polyunsaturated fatty acid supplementation in infants born at term. *Cochrane Database of Systematic Reviews*(3). https://doi.org/doi:10.1002/14651858.CD000376.pub4
127. Jin, H., Leng, Q., & Li, C. (2012). Dietary flavonoid for preventing colorectal neoplasms. *Cochrane Database of Systematic Reviews*(8). https://doi.org/doi:10.1002/14651858.CD009350.pub2
128. Jull, A. B., Ni Mhurchu, C., Bennett, D. A., Dunshea-Mooij, C. A. E., & Rodgers, A. (2008). Chitosan for overweight or obesity. *Cochrane Database of Systematic Reviews*(3). https://doi.org/10.1002/14651858.CD003892.pub3
129. Jurgens, T. M., Whelan, A. M., Killian, L., Doucette, S., Kirk, S., & Foy, E. (2012). Green tea for weight loss and weight maintenance in overweight or obese adults. *Cochrane Database of Systematic Reviews*(12). https://doi.org/10.1002/14651858.CD008650.pub2
130. Keats, E. C., Haider, B. A., Tam, E., & Bhutta, Z. A. (2019). Multiple-micronutrient supplementation for women during pregnancy. *Cochrane Database of Systematic Reviews*(3). https://doi.org/10.1002/14651858.CD004905.pub6
131. Kelly, S. A. M., Hartley, L., Loveman, E., Colquitt, J. L., Jones, H. M., Al-Khudairy, L., Clar, C., Germanò, R., Lunn, H. R., Frost, G., & et al. (2017). Whole grain cereals for the primary or secondary prevention of cardiovascular disease. *Cochrane Database of Systematic Reviews*(8). https://doi.org/10.1002/14651858.CD005051.pub3
132. Kendrick, D., Kumar, A., Carpenter, H., Zijlstra, G. A. R., Skelton, D. A., Cook, J. R., Stevens, Z., Belcher, C. M., Haworth, D., Gawler, S. J., & et al. (2014). Exercise for reducing fear of falling in older people living in the community. *Cochrane Database of Systematic Reviews*(11). https://doi.org/10.1002/14651858.CD009848.pub2
133. Kongwattanakul, K., Duangkum, C., Ngamjarus, C., Lumbiganon, P., Cuthbert, A., Weeks, J., & Sothornwit, J. (2024). Calcium supplementation (other than for preventing or treating hypertension) for improving pregnancy and infant outcomes. *Cochrane Database of Systematic Reviews*(11). https://doi.org/10.1002/14651858.CD007079.pub4
134. Kramer, M., & Kakuma, R. (2012). Maternal dietary antigen avoidance during pregnancy or lactation, or both, for preventing or treating atopic disease in the child. *Cochrane Database of Systematic Reviews*(9). https://doi.org/doi:10.1002/14651858.CD000133.pub3
135. Kramer, M., & Kakuma, R. (2012). Optimal duration of exclusive breastfeeding. *Cochrane Database of Systematic Reviews*(8). https://doi.org/doi:10.1002/14651858.CD003517.pub2
136. Kramer, M. S., & McDonald, S. W. (2006). Aerobic exercise for women during pregnancy. *Cochrane Database of Systematic Reviews*(3). https://doi.org/10.1002/14651858.CD000180.pub2
137. Kristjansson, E., Dignam, M., Rizvi, A., Osman, M., Magwood, O., Olarte, D., Cohen, J. F. W., Krasevec, J., Grover, T., Labelle, P. R., & et al. (2025). School feeding programs for improving the physical and psychological health of school children experiencing socioeconomic disadvantage. *Cochrane Database of Systematic Reviews*(12). https://doi.org/10.1002/14651858.CD014794.pub2

138. Kristjansson, E., Francis, D., Liberato, S., Benkhalti Jandu, M., Welch, V., Batal, M., Greenhalgh, T., Rader, T., Noonan, E., Shea, B., & et al. (2015). Food supplementation for improving the physical and psychosocial health of socio-economically disadvantaged children aged three months to five years. *Cochrane Database of Systematic Reviews*(3). https://doi.org/doi:10.1002/14651858.CD009924.pub2
139. Langford, R., Bonell, C. P., Jones, H. E., Pouliou, T., Murphy, S. M., Waters, E., Komro, K. A., Gibbs, L. F., Magnus, D., & Campbell, R. (2014). The WHO Health Promoting School framework for improving the health and well-being of students and their academic achievement. *Cochrane Database of Systematic Reviews*(4). https://doi.org/10.1002/14651858.CD008958.pub2
140. Larun, L., Nordheim, L. V., Ekeland, E., Hagen, K. B., & Heian, F. (2006). Exercise in prevention and treatment of anxiety and depression among children and young people. *Cochrane Database of Systematic Reviews*(3). https://doi.org/10.1002/14651858.CD004691.pub2
141. Lassi, Z. S., Kurji, J., Oliveira, C. S. D., Moin, A., & Bhutta, Z. A. (2020). Zinc supplementation for the promotion of growth and prevention of infections in infants less than six months of age. *Cochrane Database of Systematic Reviews*(4). https://doi.org/10.1002/14651858.CD010205.pub2
142. Lassi, Z. S., Moin, A., & Bhutta, Z. A. (2016). Zinc supplementation for the prevention of pneumonia in children aged 2 months to 59 months. *Cochrane Database of Systematic Reviews*(12). https://doi.org/10.1002/14651858.CD005978.pub3
143. Lassi, Z. S., Salam, R. A., Haider, B. A., & Bhutta, Z. A. (2013). Folic acid supplementation during pregnancy for maternal health and pregnancy outcomes. *Cochrane Database of Systematic Reviews*(3). https://doi.org/10.1002/14651858.CD006896.pub2
144. Lawrenson, J., & Evans, J. (2015). Omega 3 fatty acids for preventing or slowing the progression of age-related macular degeneration. *Cochrane Database of Systematic Reviews*(4). https://doi.org/doi:10.1002/14651858.CD010015.pub3
145. Lazzerini, M., Rubert, L., & Pani, P. (2013). Specially formulated foods for treating children with moderate acute malnutrition in low- and middle-income countries. *Cochrane Database of Systematic Reviews*(6). https://doi.org/doi:10.1002/14651858.CD009584.pub2
146. Lee, D. C. W., O'Brien, K. M., McCrabb, S., Wolfenden, L., Tzelepis, F., Barnes, C., Yoong, S., Bartlem, K. M., & Hodder, R. K. (2024). Strategies for enhancing the implementation of school-based policies or practices targeting diet, physical activity, obesity, tobacco or alcohol use. *Cochrane Database of Systematic Reviews*(12). https://doi.org/10.1002/14651858.CD011677.pub4
147. Lerch, C., & Meissner, T. (2007). Interventions for the prevention of nutritional rickets in term born children. *Cochrane Database of Systematic Reviews*(4). https://doi.org/doi:10.1002/14651858.CD006164.pub2
148. Lhachimi, S. K., Pega, F., Heise, T. L., Fenton, C., Gartlehner, G., Griebler, U., Sommer, I., Bombana, M., & Katikireddi, S. (2020). Taxation of the fat content of foods for reducing their consumption and preventing obesity or other adverse health outcomes. *Cochrane Database of Systematic Reviews*(9). https://doi.org/10.1002/14651858.CD012415.pub2
149. Liddle, S., & Pennick, V. (2015). Interventions for preventing and treating low-back and pelvic pain during pregnancy. *Cochrane Database of Systematic Reviews*(9). https://doi.org/doi:10.1002/14651858.CD001139.pub4
150. Liu, C. J., & Latham, N. K. (2009). Progressive resistance strength training for improving physical function in older adults. *Cochrane Database of Systematic Reviews*(3). https://doi.org/10.1002/14651858.CD002759.pub2
151. Loveman, E., Al-Khudairy, L., Johnson, R., Robertson, W., Colquitt, J., Mead, E., Ells, L., Metzendorf, M., & Rees, K. (2015). Parent-only interventions for childhood overweight or obesity in children aged 5 to 11 years. *Cochrane Database of Systematic Reviews*(12). https://doi.org/doi:10.1002/14651858.CD012008
152. Low, M. S. Y., Speedy, J., Styles, C. E., De-Regil, L. M., & Pasricha, S. R. (2016). Daily iron supplementation for improving anaemia, iron status and health in menstruating women. *Cochrane Database of Systematic Reviews*(4). https://doi.org/10.1002/14651858.CD009747.pub2

153. Lumbiganon, P., Martis, R., Laopaiboon, M., Festin, M., Ho, J., & Hakimi, M. (2016). Antenatal breastfeeding education for increasing breastfeeding duration. *Cochrane Database of Systematic Reviews*(12). https://doi.org/doi:10.1002/14651858.CD006425.pub4
154. Maas, T., Kaper, J., Sheikh, A., Knottnerus, J., Wesseling, G., Dompeling, E., Muris, J., & van Schayck, C. (2009). Mono and multifaceted inhalant and/or food allergen reduction interventions for preventing asthma in children at high risk of developing asthma. *Cochrane Database of Systematic Reviews*(3). https://doi.org/doi:10.1002/14651858.CD006480.pub2
155. Makrides, M., Crosby, D. D., Shepherd, E., & Crowther, C. A. (2014). Magnesium supplementation in pregnancy. *Cochrane Database of Systematic Reviews*(4). https://doi.org/10.1002/14651858.CD000937.pub2
156. Malouf, R., & Grimley Evans, J. (2008). Folic acid with or without vitamin B12 for the prevention and treatment of healthy elderly and demented people. *Cochrane Database of Systematic Reviews*(4). https://doi.org/doi:10.1002/14651858.CD004514.pub2
157. Martin, A., Booth, J. N., Laird, Y., Sproule, J., Reilly, J. J., & Saunders, D. H. (2018). Physical activity, diet and other behavioural interventions for improving cognition and school achievement in children and adolescents with obesity or overweight. *Cochrane Database of Systematic Reviews*(3). https://doi.org/10.1002/14651858.CD009728.pub4
158. Martin, N., Germanò, R., Hartley, L., Adler, A. J., & Rees, K. (2015). Nut consumption for the primary prevention of cardiovascular disease. *Cochrane Database of Systematic Reviews*(9). https://doi.org/10.1002/14651858.CD011583.pub2
159. Mathew, M., Ervin, A., Tao, J., & Davis, R. (2012). Antioxidant vitamin supplementation for preventing and slowing the progression of age-related cataract. *Cochrane Database of Systematic Reviews*(6). https://doi.org/doi:10.1002/14651858.CD004567.pub2
160. McCauley, M. E., van den Broek, N., Dou, L., & Othman, M. (2015). Vitamin A supplementation during pregnancy for maternal and newborn outcomes. *Cochrane Database of Systematic Reviews*(10). https://doi.org/10.1002/14651858.CD008666.pub3
161. McCleery, J., Abraham, R., Denton, D., Rutjes, A., Chong, L., Al-Assaf, A., Griffith, D., Rafeeq, S., Yaman, H., Malik, M., & et al. (2018). Vitamin and mineral supplementation for preventing dementia or delaying cognitive decline in people with mild cognitive impairment. *Cochrane Database of Systematic Reviews*(11). https://doi.org/doi:10.1002/14651858.CD011905.pub2
162. McLaren, L., Sumar, N., Barberio, A. M., Trieu, K., Lorenzetti, D. L., Tarasuk, V., Webster, J., & Campbell, N. R. C. (2016). Population-level interventions in government jurisdictions for dietary sodium reduction. *Cochrane Database of Systematic Reviews*(9). https://doi.org/10.1002/14651858.CD010166.pub2
163. Mead, E., Brown, T., Rees, K., Azevedo, L., Whittaker, V., Jones, D., Olajide, J., Mainardi, G., Corpeleijn, E., O'Malley, C., & et al. (2017). Diet, physical activity and behavioural interventions for the treatment of overweight or obese children from the age of 6 to 11 years. *Cochrane Database of Systematic Reviews*(6). https://doi.org/doi:10.1002/14651858.CD012651
164. Meher, S., & Duley, L. (2006). Exercise or other physical activity for preventing pre-eclampsia and its complications. *Cochrane Database of Systematic Reviews*(2). https://doi.org/10.1002/14651858.CD005942
165. Meher, S., & Duley, L. (2006). Garlic for preventing pre-eclampsia and its complications. *Cochrane Database of Systematic Reviews*(3). https://doi.org/10.1002/14651858.CD006065
166. Metzendorf, M.-I., Wieland, L., & Richter, B. (2024). Mobile health (m-health) smartphone interventions for adolescents and adults with overweight or obesity. *Cochrane Database of Systematic Reviews*(2). https://doi.org/doi:10.1002/14651858.CD013591.pub2
167. Middleton, P., Gomersall, J. C., Gould, J. F., Shepherd, E., Olsen, S. F., & Makrides, M. (2018). Omega-3 fatty acid addition during pregnancy. *Cochrane Database of Systematic Reviews*(11). https://doi.org/10.1002/14651858.CD003402.pub3

168. Miller, B. J., Murray, L., Beckmann, M. M., Kent, T., & Macfarlane, B. (2013). Dietary supplements for preventing postnatal depression. *Cochrane Database of Systematic Reviews*(10). https://doi.org/10.1002/14651858.CD009104.pub2
169. Milne, A., Potter, J., Vivanti, A., & Avenell, A. (2009). Protein and energy supplementation in elderly people at risk from malnutrition. *Cochrane Database of Systematic Reviews*(2). https://doi.org/doi:10.1002/14651858.CD003288.pub3
170. Montgomery, P., & Dennis, J. (2002). Physical exercise for sleep problems in adults aged 60+. *Cochrane Database of Systematic Reviews*(4). https://doi.org/doi:10.1002/14651858.CD003404
171. Morgan, E., Schoonees, A., Sriram, U., Faure, M., & Seguin-Fowler, R. (2020). Caregiver involvement in interventions for improving children's dietary intake and physical activity behaviors. *Cochrane Database of Systematic Reviews*(1). https://doi.org/doi:10.1002/14651858.CD012547.pub2
172. Mosdøl, A., Lidal, I. B., Straumann, G. H., & Vist, G. E. (2017). Targeted mass media interventions promoting healthy behaviours to reduce risk of non-communicable diseases in adult, ethnic minorities. *Cochrane Database of Systematic Reviews*(2). https://doi.org/10.1002/14651858.CD011683.pub2
173. Motuhifonua, S., Lin, L., Alsweiler, J., Crawford, T., & Crowther, C. (2023). Antenatal dietary supplementation with myo-inositol for preventing gestational diabetes. *Cochrane Database of Systematic Reviews*(2). https://doi.org/doi:10.1002/14651858.CD011507.pub3
174. Muktabhant, B., Lawrie, T. A., Lumbiganon, P., & Laopaiboon, M. (2015). Diet or exercise, or both, for preventing excessive weight gain in pregnancy. *Cochrane Database of Systematic Reviews*(6). https://doi.org/10.1002/14651858.CD007145.pub3
175. Murtagh, E. M., Murphy, M. H., Milton, K., Roberts, N. W., O'Gorman, C. S. M., & Foster, C. (2020). Interventions outside the workplace for reducing sedentary behaviour in adults under 60 years of age. *Cochrane Database of Systematic Reviews*(7). https://doi.org/10.1002/14651858.CD012554.pub2
176. Naude, C. E., Brand, A., Schoonees, A., Nguyen, K. A., Chaplin, M., & Volmink, J. (2022). Low-carbohydrate versus balanced-carbohydrate diets for reducing weight and cardiovascular risk. *Cochrane Database of Systematic Reviews*(1). https://doi.org/10.1002/14651858.CD013334.pub2
177. Naude, C. E., Visser, M. E., Nguyen, K. A., Durao, S., & Schoonees, A. (2018). Effects of total fat intake on bodyweight in children. *Cochrane Database of Systematic Reviews*(7). https://doi.org/10.1002/14651858.CD012960.pub2
178. Nault, D., Machingo, T. A., Shipper, A. G., Antiporta, D. A., Hamel, C., Nourouzpour, S., Konstantinidis, M., Phillips, E., Lipski, E. A., & Wieland, L. S. (2024). Zinc for prevention and treatment of the common cold. *Cochrane Database of Systematic Reviews*(5). https://doi.org/10.1002/14651858.CD014914.pub2
179. Ndikom, C., Fawole, B., & Ilesanmi, R. (2014). Extra fluids for breastfeeding mothers for increasing milk production. *Cochrane Database of Systematic Reviews*(6). https://doi.org/doi:10.1002/14651858.CD008758.pub2
180. Neil-Sztramko, S. E., Caldwell, H., & Dobbins, M. (2021). School-based physical activity programs for promoting physical activity and fitness in children and adolescents aged 6 to 18. *Cochrane Database of Systematic Reviews*(9). https://doi.org/10.1002/14651858.CD007651.pub3
181. Odigwe, C. C., Smedslund, G., Ejemot-Nwadiaro, R. I., Anyanechi, C. C., & Krawinkel, M. B. (2010). Supplementary vitamin E, selenium, cysteine and riboflavin for preventing kwashiorkor in preschool children in developing countries. *Cochrane Database of Systematic Reviews*(4). https://doi.org/10.1002/14651858.CD008147.pub2
182. Ojha, S., Elfzzani, Z., Kwok, T. C., & Dorling, J. (2020). Education of family members to support weaning to solids and nutrition in later infancy in term-born infants. *Cochrane Database of Systematic Reviews*(7). https://doi.org/10.1002/14651858.CD012241.pub2
183. Oliveira, J. M., Allert, R., & East, C. E. (2016). Vitamin A supplementation for postpartum women. *Cochrane Database of Systematic Reviews*(3). https://doi.org/10.1002/14651858.CD005944.pub3

184. Ong, T. G., Gordon, M., Banks, S. S. C., Thomas, M. R., & Akobeng, A. K. (2019). Probiotics to prevent infantile colic. *Cochrane Database of Systematic Reviews*(3). https://doi.org/10.1002/14651858.CD012473.pub2
185. Ooi, C., Loke, S., Yassin, Z., & Hamid, T. (2011). Carbohydrates for improving the cognitive performance of independent-living older adults with normal cognition or mild cognitive impairment. *Cochrane Database of Systematic Reviews*(4). https://doi.org/doi:10.1002/14651858.CD007220.pub2
186. Osborn, D., & Sinn, J. (2006). Soy formula for prevention of allergy and food intolerance in infants. *Cochrane Database of Systematic Reviews*(4). https://doi.org/doi:10.1002/14651858.CD003741.pub4
187. Osborn, D., & Sinn, J. (2013). Prebiotics in infants for prevention of allergy. *Cochrane Database of Systematic Reviews*(3). https://doi.org/doi:10.1002/14651858.CD006474.pub3
188. Osborn, D., Sinn, J., & Jones, L. (2018). Infant formulas containing hydrolysed protein for prevention of allergic disease. *Cochrane Database of Systematic Reviews*(10). https://doi.org/doi:10.1002/14651858.CD003664.pub6
189. Ota, E., da Silva Lopes, K., Middleton, P., Flenady, V., Wariki, W., Rahman, M., Tobe-Gai, R., & Mori, R. (2020). Antenatal interventions for preventing stillbirth, fetal loss and perinatal death: an overview of Cochrane systematic reviews. *Cochrane Database of Systematic Reviews*(12). https://doi.org/doi:10.1002/14651858.CD009599.pub2
190. Ota, E., Hori, H., Mori, R., Tobe-Gai, R., & Farrar, D. (2015). Antenatal dietary education and supplementation to increase energy and protein intake. *Cochrane Database of Systematic Reviews*(6). https://doi.org/doi:10.1002/14651858.CD000032.pub3
191. Padhani, Z., Moazzam, Z., Ashraf, A., Bilal, H., Salam, R., Das, J., & Bhutta, Z. (2021). Vitamin C supplementation for prevention and treatment of pneumonia. *Cochrane Database of Systematic Reviews*(11). https://doi.org/doi:10.1002/14651858.CD013134.pub3
192. Palacios, C., Kostiuk, L. L., Cuthbert, A., & Weeks, J. (2024). Vitamin D supplementation for women during pregnancy. *Cochrane Database of Systematic Reviews*(7). https://doi.org/10.1002/14651858.CD008873.pub5
193. Palmer, M., Henschke, N., Bergman, H., Villanueva, G., Maayan, N., Tamrat, T., Mehl, G., Glenton, C., Lewin, S., Fønhus, M., & et al. (2020). Targeted client communication via mobile devices for improving maternal, neonatal, and child health. *Cochrane Database of Systematic Reviews*(8). https://doi.org/doi:10.1002/14651858.CD013679
194. Parry, S. P., Coenen, P., Shrestha, N., O'Sullivan, P. B., Maher, C. G., & Straker, L. M. (2019). Workplace interventions for increasing standing or walking for decreasing musculoskeletal symptoms in sedentary workers. *Cochrane Database of Systematic Reviews*(11). https://doi.org/10.1002/14651858.CD012487.pub2
195. Patchen, B. K., Best, C. M., Boiteau, J., Solvik, B. S., Vonderschmidt, A., Xu, J., Cohen, R. T., & Cassano, P. A. (2025). Vitamin D supplementation in pregnant or breastfeeding women or young children for preventing asthma. *Cochrane Database of Systematic Reviews*(8). https://doi.org/10.1002/14651858.CD013396.pub2
196. Peña-Rosas, J., Mithra, P., Unnikrishnan, B., Kumar, N., De-Regil, L., Nair, N., Garcia-Casal, M., & Solon, J. (2019). Fortification of rice with vitamins and minerals for addressing micronutrient malnutrition. *Cochrane Database of Systematic Reviews*(10). https://doi.org/doi:10.1002/14651858.CD009902.pub2
197. Peña-Rosas, J. P., De-Regil, L. M., Gomez Malave, H., Flores-Urrutia, M. C., & Dowswell, T. (2015). Intermittent oral iron supplementation during pregnancy. *Cochrane Database of Systematic Reviews*(10). https://doi.org/10.1002/14651858.CD009997.pub2
198. Petkovic, J., Duench, S., Trawin, J., Dewidar, O., Pardo Pardo, J., Simeon, R., DesMeules, M., Gagnon, D., Hatcher Roberts, J., Hossain, A., & et al. (2021). Behavioural interventions delivered through interactive social media for health behaviour change, health outcomes, and health equity in the adult population. *Cochrane Database of Systematic Reviews*(5). https://doi.org/doi:10.1002/14651858.CD012932.pub2

199. Pfinder, M., Heise, T. L., Hilton Boon, M., Pega, F., Fenton, C., Griebler, U., Gartlehner, G., Sommer, I., Katikireddi, S. V., & Lhachimi, S. K. (2020). Taxation of unprocessed sugar or sugar-added foods for reducing their consumption and preventing obesity or other adverse health outcomes. *Cochrane Database of Systematic Reviews*(4). https://doi.org/10.1002/14651858.CD012333.pub2
200. Phillips, S. M., Spiga, F., Moore, T. H. M., Dawson, S., Stockton, H., Rizk, R., Cheng, H. Y., Hodder, R. K., Gao, Y., Hillier-Brown, F., & et al. (2025). Interventions to prevent obesity in children aged 2 to 4 years old. *Cochrane Database of Systematic Reviews*(6). https://doi.org/10.1002/14651858.CD015326.pub2
201. Priebe, M., van Binsbergen, J., de Vos, R., & Vonk, R. J. (2008). Whole grain foods for the prevention of type 2 diabetes mellitus. *Cochrane Database of Systematic Reviews*(1). https://doi.org/10.1002/14651858.CD006061.pub2
202. Priest, N., Armstrong, R., Doyle, J., & Waters, E. (2008). Policy interventions implemented through sporting organisations for promoting healthy behaviour change. *Cochrane Database of Systematic Reviews*(3). https://doi.org/10.1002/14651858.CD004809.pub3
203. Rees, K., Al-Khudairy, L., Takeda, A., & Stranges, S. (2021). Vegan dietary pattern for the primary and secondary prevention of cardiovascular diseases. *Cochrane Database of Systematic Reviews*(2). https://doi.org/10.1002/14651858.CD013501.pub2
204. Rees, K., Dyakova, M., Wilson, N., Ward, K., Thorogood, M., & Brunner, E. (2013). Dietary advice for reducing cardiovascular risk. *Cochrane Database of Systematic Reviews*(12). https://doi.org/10.1002/14651858.CD002128.pub5
205. Rees, K., Hartley, L., Day, C., Flowers, N., Clarke, A., & Stranges, S. (2013). Selenium supplementation for the primary prevention of cardiovascular disease. *Cochrane Database of Systematic Reviews*(1). https://doi.org/doi:10.1002/14651858.CD009671.pub2
206. Rees, K., Takeda, A., Martin, N., Ellis, L., Wijesekara, D., Vepa, A., Das, A., Hartley, L., & Stranges, S. (2019). Mediterranean-style diet for the primary and secondary prevention of cardiovascular disease. *Cochrane Database of Systematic Reviews*(3). https://doi.org/10.1002/14651858.CD009825.pub3
207. Richards, J., Hillsdon, M., Thorogood, M., & Foster, C. (2013). Face-to-face interventions for promoting physical activity. *Cochrane Database of Systematic Reviews*(9). https://doi.org/10.1002/14651858.CD010392.pub2
208. Richards, J., Thorogood, M., Hillsdon, M., & Foster, C. (2013). Face-to-face versus remote and web 2.0 interventions for promoting physical activity. *Cochrane Database of Systematic Reviews*(9). https://doi.org/10.1002/14651858.CD010393.pub2
209. Riley, P., Moore, D., Ahmed, F., Sharif, M., & Worthington, H. (2015). Xylitol-containing products for preventing dental caries in children and adults. *Cochrane Database of Systematic Reviews*(3). https://doi.org/doi:10.1002/14651858.CD010743.pub2
210. Rumbold, A., Ota, E., Hori, H., Miyazaki, C., & Crowther, C. A. (2015). Vitamin E supplementation in pregnancy. *Cochrane Database of Systematic Reviews*(9). https://doi.org/10.1002/14651858.CD004069.pub3
211. Rumbold, A., Ota, E., Nagata, C., Shahrook, S., & Crowther, C. A. (2015). Vitamin C supplementation in pregnancy. *Cochrane Database of Systematic Reviews*(9). https://doi.org/10.1002/14651858.CD004072.pub3
212. Rutjes, A. W. S., Denton, D. A., Di Nisio, M., Chong, L. Y., Abraham, R. P., Al-Assaf, A. S., Anderson, J. L., Malik, M. A., Vernooij, R. W. M., Martínez, G., & et al. (2018). Vitamin and mineral supplementation for maintaining cognitive function in cognitively healthy people in mid and late life. *Cochrane Database of Systematic Reviews*(12). https://doi.org/10.1002/14651858.CD011906.pub2
213. Salam, R. A., Zuberi, N. F., & Bhutta, Z. A. (2015). Pyridoxine (vitamin B6) supplementation during pregnancy or labour for maternal and neonatal outcomes. *Cochrane Database of Systematic Reviews*(6). https://doi.org/10.1002/14651858.CD000179.pub3

214. Santos, J., Christoforou, A., Trieu, K., McKenzie, B., Downs, S., Billot, L., Webster, J., & Li, M. (2019). Iodine fortification of foods and condiments, other than salt, for preventing iodine deficiency disorders. *Cochrane Database of Systematic Reviews*(2). https://doi.org/doi:10.1002/14651858.CD010734.pub2
215. Schindler, T., Sinn, J., & Osborn, D. (2016). Polyunsaturated fatty acid supplementation in infancy for the prevention of allergy. *Cochrane Database of Systematic Reviews*(10). https://doi.org/doi:10.1002/14651858.CD010112.pub2
216. Schmucker, C., Eisele-Metzger, A., Meerpohl, J. J., Lehane, C., Kuellenberg de Gaudry, D., Lohner, S., & Schwingshackl, L. (2022). Effects of a gluten-reduced or gluten-free diet for the primary prevention of cardiovascular disease. *Cochrane Database of Systematic Reviews*(2). https://doi.org/10.1002/14651858.CD013556.pub2
217. Schwenger, E. M., Tejani, A. M., & Loewen, P. S. (2015). Probiotics for preventing urinary tract infections in adults and children. *Cochrane Database of Systematic Reviews*(12). https://doi.org/10.1002/14651858.CD008772.pub2
218. Scott, A. M., Clark, J., Julien, B., Islam, F., Roos, K., Grimwood, K., Little, P., & Del Mar, C. B. (2019). Probiotics for preventing acute otitis media in children. *Cochrane Database of Systematic Reviews*(6). https://doi.org/10.1002/14651858.CD012941.pub2
219. Seron, P., Lanas, F., Pardo Hernandez, H., & Bonfill Cosp, X. (2014). Exercise for people with high cardiovascular risk. *Cochrane Database of Systematic Reviews*(8). https://doi.org/10.1002/14651858.CD009387.pub2
220. Sguassero, Y., de Onis, M., Bonotti, A. M., & Carroli, G. (2012). Community-based supplementary feeding for promoting the growth of children under five years of age in low and middle income countries. *Cochrane Database of Systematic Reviews*(6). https://doi.org/10.1002/14651858.CD005039.pub3
221. Shah, D., Sachdev, H. S., Gera, T., De-Regil, L. M., & Peña-Rosas, J. P. (2016). Fortification of staple foods with zinc for improving zinc status and other health outcomes in the general population. *Cochrane Database of Systematic Reviews*(6). https://doi.org/10.1002/14651858.CD010697.pub2
222. Shaw, K., Gennat, H., O'Rourke, P., & Del Mar, C. (2006). Exercise for overweight or obesity. *Cochrane Database of Systematic Reviews*(4). https://doi.org/doi:10.1002/14651858.CD003817.pub3
223. Shepherd, E., Gomersall, J. C., Tieu, J., Han, S., Crowther, C. A., & Middleton, P. (2017). Combined diet and exercise interventions for preventing gestational diabetes mellitus. *Cochrane Database of Systematic Reviews*(11). https://doi.org/10.1002/14651858.CD010443.pub3
224. Sherrington, C., Fairhall, N. J., Wallbank, G. K., Tiedemann, A., Michaleff, Z. A., Howard, K., Clemson, L., Hopewell, S., & Lamb, S. E. (2019). Exercise for preventing falls in older people living in the community. *Cochrane Database of Systematic Reviews*(1). https://doi.org/10.1002/14651858.CD012424.pub2
225. Showell, M., Mackenzie-Proctor, R., Jordan, V., & Hart, R. (2020). Antioxidants for female subfertility. *Cochrane Database of Systematic Reviews*(8). https://doi.org/doi:10.1002/14651858.CD007807.pub4
226. Shrestha, N., Kukkonen-Harjula, K. T., Verbeek, J. H., Ijaz, S., Hermans, V., & Pedisic, Z. (2018). Workplace interventions for reducing sitting at work. *Cochrane Database of Systematic Reviews*(12). https://doi.org/10.1002/14651858.CD010912.pub5
227. Smith, H. A., & Becker, G. E. (2016). Early additional food and fluids for healthy breastfed full-term infants. *Cochrane Database of Systematic Reviews*(8). https://doi.org/10.1002/14651858.CD006462.pub4
228. Spiga, F., Davies, A. L., Tomlinson, E., Moore, T. H. M., Dawson, S., Breheny, K., Savović, J., Gao, Y., Phillips, S. M., Hillier-Brown, F., & et al. (2024). Interventions to prevent obesity in children aged 5 to 11 years old. *Cochrane Database of Systematic Reviews*(5). https://doi.org/10.1002/14651858.CD015328.pub2
229. Spiga, F., Tomlinson, E., Davies, A. L., Moore, T. H. M., Dawson, S., Breheny, K., Savović, J., Hodder, R. K., Wolfenden, L., Higgins, J. P. T., & et al. (2024). Interventions to prevent obesity in children aged 12 to 18 years old. *Cochrane Database of Systematic Reviews*(5). https://doi.org/10.1002/14651858.CD015330.pub2

230. Suchdev, P. S., Jefferds, M. E. D., Ota, E., da Silva Lopes, K., & De-Regil, L. M. (2020). Home fortification of foods with multiple micronutrient powders for health and nutrition in children under two years of age. *Cochrane Database of Systematic Reviews*(2). https://doi.org/10.1002/14651858.CD008959.pub3
231. Suchdev, P. S., Peña-Rosas, J. P., & De-Regil, L. M. (2015). Multiple micronutrient powders for home (point-of-use) fortification of foods in pregnant women. *Cochrane Database of Systematic Reviews*(6). https://doi.org/10.1002/14651858.CD011158.pub2
232. Sydenham, E., Dangour, A. D., & Lim, W. S. (2012). Omega 3 fatty acid for the prevention of cognitive decline and dementia. *Cochrane Database of Systematic Reviews*(6). https://doi.org/10.1002/14651858.CD005379.pub3
233. Takahashi, R., Ota, E., Hoshi, K., Naito, T., Toyoshima, Y., Yuasa, H., Mori, R., & Nango, E. (2017). Fluoride supplementation (with tablets, drops, lozenges or chewing gum) in pregnant women for preventing dental caries in the primary teeth of their children. *Cochrane Database of Systematic Reviews*(10). https://doi.org/10.1002/14651858.CD011850.pub2
234. Tan, M. L., Abrams, S. A., & Osborn, D. A. (2020). Vitamin D supplementation for term breastfed infants to prevent vitamin D deficiency and improve bone health. *Cochrane Database of Systematic Reviews*(12). https://doi.org/10.1002/14651858.CD013046.pub2
235. Thompson, R. L., Summerbell, C. D., Hooper, L., Higgins, J. P. T., Little, P., Talbot, D., & Ebrahim, S. (2003). Dietary advice given by a dietitian versus other health professional or self-help resources to reduce blood cholesterol. *Cochrane Database of Systematic Reviews*(3). https://doi.org/10.1002/14651858.CD001366
236. Tieu, J., Shepherd, E., Middleton, P., & Crowther, C. A. (2017). Dietary advice interventions in pregnancy for preventing gestational diabetes mellitus. *Cochrane Database of Systematic Reviews*(1). https://doi.org/10.1002/14651858.CD006674.pub3
237. Tubert-Jeannin, S., Auclair, C., Amsallem, E., Tramini, P., Gerbaud, L., Ruffieux, C., Schulte, A. G., Koch, M. J., Rège-Walther, M., & Ismail, A. (2011). Fluoride supplements (tablets, drops, lozenges or chewing gums) for preventing dental caries in children. *Cochrane Database of Systematic Reviews*(12). https://doi.org/10.1002/14651858.CD007592.pub2
238. Turawa, E., Musekiwa, A., & Rohwer, A. (2020). Interventions for preventing postpartum constipation. *Cochrane Database of Systematic Reviews*(8). https://doi.org/doi:10.1002/14651858.CD011625.pub3
239. Uthman, O., Hartley, L., Rees, K., Taylor, F., Ebrahim, S., & Clarke, A. (2015). Multiple risk factor interventions for primary prevention of cardiovascular disease in low- and middle-income countries. *Cochrane Database of Systematic Reviews*(8). https://doi.org/doi:10.1002/14651858.CD011163.pub2
240. Valentín-Gudiol, M., Mattern-Baxter, K., Girabent-Farrés, M., Bagur-Calafat, C., Hadders-Algra, M., & Angulo-Barroso, R. (2017). Treadmill interventions in children under six years of age at risk of neuromotor delay. *Cochrane Database of Systematic Reviews*(7). https://doi.org/doi:10.1002/14651858.CD009242.pub3
241. Verhagen, A., Bierma-Zeinstra, S., Burdorf, A., Stynes, S., de Vet, H., & Koes, B. (2013). Conservative interventions for treating work-related complaints of the arm, neck or shoulder in adults. *Cochrane Database of Systematic Reviews*(12). https://doi.org/doi:10.1002/14651858.CD008742.pub2
242. Vinceti, M., Filippini, T., Del Giovane, C., Dennert, G., Zwahlen, M., Brinkman, M., Zeegers, M. P. A., Horneber, M., D'Amico, R., & Crespi, C. M. (2018). Selenium for preventing cancer. *Cochrane Database of Systematic Reviews*(1). https://doi.org/10.1002/14651858.CD005195.pub4
243. Virgara, R., Phillips, A., Lewis, L. K., Baldock, K., Wolfenden, L., Ferguson, T., Richardson, M., Okely, A., Beets, M., & Maher, C. (2021). Interventions in outside-school hours childcare settings for promoting physical activity amongst schoolchildren aged 4 to 12 years. *Cochrane Database of Systematic Reviews*(9). https://doi.org/10.1002/14651858.CD013380.pub2
244. Visser, J., McLachlan, M., Maayan, N., & Garner, P. (2018). Community-based supplementary feeding for food insecure, vulnerable and malnourished populations – an overview of systematic reviews. *Cochrane Database of Systematic Reviews*(11). https://doi.org/doi:10.1002/14651858.CD010578.pub2

245. Visser, M. E., Schoonees, A., Ezekiel, C. N., Randall, N. P., & Naude, C. E. (2020). Agricultural and nutritional education interventions for reducing aflatoxin exposure to improve infant and child growth in low- and middle-income countries. *Cochrane Database of Systematic Reviews*(4). https://doi.org/10.1002/14651858.CD013376.pub2
246. von Philipsborn, P., Stratil, J. M., Burns, J., Busert, L. K., Pfadenhauer, L. M., Polus, S., Holzapfel, C., Hauner, H., & Rehfuess, E. (2019). Environmental interventions to reduce the consumption of sugar-sweetened beverages and their effects on health. *Cochrane Database of Systematic Reviews*(6). https://doi.org/10.1002/14651858.CD012292.pub2
247. Wang, H. Z., Hayles, E. H., Fiander, M., Sinn, J. K. H., & Osborn, D. A. (2025). Probiotics in infants for prevention of allergic disease. *Cochrane Database of Systematic Reviews*(6). https://doi.org/10.1002/14651858.CD006475.pub3
248. Weingarten, M., Zalmanovici Trestioreanu, A., & Yaphe, J. (2008). Dietary calcium supplementation for preventing colorectal cancer and adenomatous polyps. *Cochrane Database of Systematic Reviews*(1). https://doi.org/doi:10.1002/14651858.CD003548.pub4
249. Wieland, L., Falzon, L., Sciamanna, C., Trudeau, K., Brodney Folse, S., Schwartz, J., & Davidson, K. (2012). Interactive computer-based interventions for weight loss or weight maintenance in overweight or obese people. *Cochrane Database of Systematic Reviews*(8). https://doi.org/doi:10.1002/14651858.CD007675.pub2
250. Wieland, L., Skoetz, N., Pilkington, K., Harbin, S., Vempati, R., & Berman, B. (2022). Yoga for chronic non-specific low back pain. *Cochrane Database of Systematic Reviews*(11). https://doi.org/doi:10.1002/14651858.CD010671.pub3
251. Winzenberg, T. M., Powell, S., Shaw, K. A., & Jones, G. (2010). Vitamin D supplementation for improving bone mineral density in children. *Cochrane Database of Systematic Reviews*(10). https://doi.org/10.1002/14651858.CD006944.pub2
252. Winzenberg, T. M., Shaw, K. A., Fryer, J., & Jones, G. (2006). Calcium supplementation for improving bone mineral density in children. *Cochrane Database of Systematic Reviews*(2). https://doi.org/10.1002/14651858.CD005119.pub2
253. Wolfenden, L., Barnes, C., Jones, J., Finch, M., Wyse, R. J., Kingsland, M., Tzelepis, F., Grady, A., Hodder, R. K., Booth, D., & et al. (2020). Strategies to improve the implementation of healthy eating, physical activity and obesity prevention policies, practices or programmes within childcare services. *Cochrane Database of Systematic Reviews*(2). https://doi.org/10.1002/14651858.CD011779.pub3
254. Wu, T., Liu, G., Li, P., & Clar, C. (2002). Iodised salt for preventing iodine deficiency disorders. *Cochrane Database of Systematic Reviews*(3). https://doi.org/doi:10.1002/14651858.CD003204
255. Yakoob, M. Y., Salam, R. A., Khan, F. R., & Bhutta, Z. A. (2016). Vitamin D supplementation for preventing infections in children under five years of age. *Cochrane Database of Systematic Reviews*(11). https://doi.org/10.1002/14651858.CD008824.pub2
256. Yamato, T., Maher, C., Saragiotto, B., Hancock, M., Ostelo, R., Cabral, C., Menezes Costa, L., & Costa, L. (2015). Pilates for low back pain. *Cochrane Database of Systematic Reviews*(7). https://doi.org/doi:10.1002/14651858.CD010265.pub2
257. Yeung, C. A., Chong, L. Y., & Glenny, A. M. (2015). Fluoridated milk for preventing dental caries. *Cochrane Database of Systematic Reviews*(9). https://doi.org/10.1002/14651858.CD003876.pub4
258. Yoong, S. L., Lum, M., Wolfenden, L., Jackson, J., Barnes, C., Hall, A. E., McCrabb, S., Pearson, N., Lane, C., Jones, J. Z., & et al. (2023). Healthy eating interventions delivered in early childhood education and care settings for improving the diet of children aged six months to six years. *Cochrane Database of Systematic Reviews*(8). https://doi.org/10.1002/14651858.CD013862.pub3
259. Young, J., Angevaren, M., Rusted, J., & Tabet, N. (2015). Aerobic exercise to improve cognitive function in older people without known cognitive impairment. *Cochrane Database of Systematic Reviews*(4). https://doi.org/10.1002/14651858.CD005381.pub4

260. Zhao, Y., Dong, B. R., & Hao, Q. (2022). Probiotics for preventing acute upper respiratory tract infections. *Cochrane Database of Systematic Reviews*(8). https://doi.org/10.1002/14651858.CD006895.pub4

Appendix 4

Plain language summary (PLS)

English:

This study looked at 260 scientific papers. The papers were about diet and exercise in healthy people and those at risk of health problems. The goal was to understand what topics have already been covered, who was involved in creating these reviews, and how much attention they have received online and in scientific circles. Most reviews focused on diet, followed by exercise and both together. Many reviews looked at adults, children, or families, but few focused on older adults or men. Most interventions were not digital (like apps or online programmes) and did not focus specific organisations (like schools or workplaces). Only one in three reviews involved patients, doctors, or other representatives. Scientists talked more about papers on exercise in scientific papers. Papers about diet were talked about in social media. New paper should focus on older adults and men and digital tools. Involving patients, doctors, and other representatives in creating papers can help make them more useful.

German:

Diese Studie untersuchte 260 wissenschaftliche Aufsätze. Die handelten von Ernährung und Bewegung bei gesunden Menschen und bei Personen, die ein erhöhtes Risiko für gesundheitliche Probleme haben. Die meisten Aufsätze haben sich auf Ernährung konzentriert, dann auf Bewegung und einige auf beides zusammen. Viele Artikel haben Erwachsene, Kinder oder Familien betrachtet, aber wenige haben sich speziell auf ältere Menschen oder Männer bezogen. Die meisten Maßnahmen in den Studien waren nicht digital, also keine Apps oder Online-Programme. Und es gab nur wenige, die bestimmte Orte betrachteten wie Schulen, Betriebe oder andere Organisationen. Nur jeder dritte Artikel hat berichtet, dass Patient:innen, Ärzt:innen oder andere Menschen, die betroffen sind, bei der Arbeit mitgemacht haben. Wissenschaftler:innen haben sich besonders oft mit Artikeln über Bewegung beschäftigt. Aber Artikel über Ernährung wurden oft in sozialen Medien erwähnt. Zukünftig sollte mehr Forschung zu älteren Menschen und Männer und digitale Hilfsmittel gemacht werden. Wenn Patient:innen, Ärzt:innen und andere Menschen, die betroffen sind, bei der Erstellung von Forschungsarbeiten mitmachen, können sie besser in die Praxis umgesetzt werden.
